# Immunity-related genes and signaling pathways under hypoxic stresses in *Haliotis diversicolor*: a transcriptome analysis

**DOI:** 10.1038/s41598-019-56150-2

**Published:** 2019-12-24

**Authors:** Yulong Sun, Xin Zhang, Yilei Wang, Robert Day, Huiping Yang, Ziping Zhang

**Affiliations:** 1 College of Animal Science, Fujian Agriculture and Forestry University, Fuzhou, 350002 P.R. China; 2 Fisheries College, Jimei University, Xiamen, 361021 China; 3 Key Laboratory of Marine Biotechnology of Fujian Province, Institute of Oceanology, Fujian Agriculture and Forestry University, Fuzhou, 350002 P.R. China; 4 School of Biosciences, University of Melbourne, Parkville, Victoria 3010 Australia; 5 School of Forest Resources and Conservation, IFAS, University of Florida, 7922 NW 71st Street, Gainesville, FL 32615 USA

**Keywords:** Ecophysiology, Transcriptomics

## Abstract

Due to increased temperatures and aquaculture density, thermal and hypoxia stresses have become serious problems for the aquaculture of abalone *Haliotis diversicolor*. Stresses lead to immunosuppression, which can cause severe negative impacts on aquaculture farms. To study the mechanism of immunosuppression after hypoxia stress and bacterial challenge, transcriptomes of *H*. *diversicolor* hemocytes involved in immunity were profiled. A total of 307,395,572 clean reads were generated and assembled into 99,774 unigenes. KEGG analysis indicated that 225 unigenes with immunologic function were mapped into immune-related pathways. Expression of 41 unigenes measured by quantitative real-time PCR (qRT-PCR) showed consistent results with that of transcriptome analysis. When exposure challenge of *Vibrio parahaemolyticus*, it is indicated that the PI3K-AKT, MAPK, NF-κB and P53 signal pathways were involved in the hypoxia-induced immunosuppression of *H*. *diversicolor*. Furthermore, when the AKT gene (*HdAKT*) was inhibited by double-stranded RNA (dsRNA), expression levels of *HdAKT* was lower than the blank and control group in hemocytes at 4 h, 12 h and 24 h (*p* < 0.05).

## Introduction

*Haliotis diversicolor* (named ‘small abalone’ in China) is one of the most commercially important aquacultured abalone species in the coastal provinces of southern China. The abalone aquaculture industry has been threatened by deteriorating environmental conditions and infectious diseases, especially in hot summers^1^. High temperature of seawater could result in low oxygen levels, and subsequently caused changes in respiration and metabolism of marine benthos^2^. Additionally, outbreaks of diseases such as the disease caused by major pathogen *Vibrio parahaemolyticus*, could occur in hot summers and cause mass mortality of cultured abalone^3,4^. Therefore, understanding of the effects of high temperature and disease on immune functions of *H*. *diversicolor* is needed for improvement of abalone aquaculture.

Under environmental and bacterial challenges, several immune related genes were cloned in *H*. *diversicolor* and the activation of these gene were investigated^5–14^. For example, the macrophage expressed gene^7^, insulin-like growth factor binding protein 7^8^, interleukin-1 receptor-associated kinase 4^9^, macrophage migration inhibitory factor^5^, and genes correlated with the NF-κB signaling pathway^10^, HIF signaling pathway^13^, toll-like receptor signaling pathway^9^ and the PI3K-AKT signal pathway^14^ have been cloned and characterized from *H*. *diversicolor* (see Additional file 1: Table S1 for the acronyms used in this paper). Particularly, it has been proved that the NF-κB signaling pathway-related genes, heat shock responsive genes, and HIF signaling pathway-related genes were significantly up-regulated in *H*. *diversicolor* after thermal or hypoxia stress^10,11,13^. Furthermore, genes related to the PI3K-AKT signal pathway were significantly down-regulated and physiological responses were affected, leading to immunosuppression when *H*. *diversicolor* was exposed to multiple stresses^14^.Using an assay of physiological and biochemical parameters of hemocytes, immunosuppression was found to be caused by high temperature^15^. So far, many research has demonstrated that environment stresses can lead to immunosuppression in vertebrates and invertebrates^16–22^.

Our published results mentioned above have also confirmed that the immune regulatory mechanisms of *H*. *diversicolor* are activated after exposure to thermal and hypoxia stresses and bacterial challenge. Thus, multiple stressors may lead to several changes including immunosuppression in abalone. Unfortunately, our knowledge of the immune response and signaling pathways that respond to hypoxia, thermal stress and bacterial pathogens in *H*. *diversicolor* is still fragmentary.

Development of DNA sequencing technology, such as the next generation sequencing (NGS), provides high-throughput tools to analyze the expression of various genes, discover novel transcripts, and identify differentially expressed genes (DEGs). NGS also provides large sequencing datasets at an affordable price^23,24^. Using the NGS technology, transcriptome analyses of *Haliotis* species and many other aquaculture mollusks have been performed and published in the recent five years^25–41^. These studies included gene expression during the early development of *H*. *diversicolor*, transcriptome profiles of wild and cultured populations of *Haliotis midae*^31^ and expression and putative function of heat shock protein 70 (*HSP70*) genes of *Haliotis laevigata* under environmental stress^27^. Previous transcriptome analysis showed that several innate immunity related pathways, such as NF-κB signaling pathway, Toll-like receptor signaling pathway, and PI3K-AKT signaling pathway are involved in immune response to environmental stress without pathogen infection in *H*. *diversicolor*^42^. These studies led to discovery of many candidate genes and provided valuable information to understand the biological characteristics of marine mollusks. However, detailed cellular and molecular mechanisms were not clearly documented yet.

In this study, the goal was to analyze the transcriptome changes in the hemocytes of *H*. *diversicolor* after exposure to bacterial challenge with or without hypoxia to reveal the molecular mechanisms and the immune responses. The objectives were to: (1) assemble transcriptome profile through RNAseq; (2) identify the differentially expressed genes in response to bacterial challenges with hypoxia or without hypoxia; (3) validate immune related genes through qRT-PCR; (4) construct the gene networks of the signaling pathways related to immunology, and (5) evaluate the interaction among the PI3K-AKT signaling pathway-related genes through RNA inhibition of *HdAKT*. This study provided a preliminary understanding of the molecular mechanisms in *H*. *diversicolor* in immunological response to bacterial challenges and hypoxia stresses.

## Results

### Sequencing and *De novo* assembly

Fourteen cDNA libraries, including BC-0h/NC-0h, BC-4h, BC-12h, BC-24h, BC-48h, NC-4h, NC-12h, NC-24h, NC-48h, HS-0h, HS-4h, HS-12h, HS-24h and HS-48h, were constructed using Illumina HiseqTM2000 paired-end sequencing technology. The average mean value of the Q30 percentage was 95.20%. After removing the adapter sequences, ambiguous nucleotides, and low-quality sequences, a total of 307,395,572 clean reads were generated through Illumina sequencing, containing 23,778,916 reads for the blank group (BC), 20,344,880 reads for the normal condition (NC), and 23,969,486 reads for the hypoxia stress group (HS). The average percentages of GC content for the clean reads were 42.69%, 43.41% and 44.50% for the BC, NC and HS (Table 1).

**Table 1:** Assembly statistics of the transcriptome of the 14 cDNA libraries.

Sample	Before Filter Reads Num	After Filter Reads Num(%)	GC%	% ≥ Q30
BC-0h	24354936	23778916 (97.63%)	42.69%	95.85%
NC-4h	20829374	20344880 (97.67%)	43.41%	95.61%
BC-4h	20899430	20489268 (98.04%)	42.96%	95.91%
NC-12h	21340802	20737464 (97.17%)	42.96%	94.66%
BC-12h	23774786	23119858 (97.25%)	42.70%	94.91%
NC-24h	21203460	20694196 (97.6%)	45.67%	95.41%
BC-24h	21677186	21209236 (97.84%)	42.77%	95.78%
NC-48h	20800322	20154168 (96.89%)	43.06%	95.03%
BC-48h	19556898	18974014 (97.02%)	43.12%	94.83%
HS-0h	24693066	23969486 (97.07%)	44.50%	94.43%
HS-4h	19287670	18748906 (97.21%)	43.27%	95.00%
HS-12h	20653144	20167554 (97.65%)	42.83%	95.85%
HS-24h	29409970	28492076 (96.88%)	42.75%	94.76%
HS-48h	27383712	26515550 (96.83%)	42.91%	94.80%

The 14 cDNA libraries (BC-0h, BC-4h, BC-12h, BC-24h, BC-48h, NC-4h, NC-12h, NC-24h, NC-48h, HS-0h, HS-4h, HS-12h, HS-24h and HS-48h) were assembled by using the Trinity software package. BC: Blank control group, normal conditions; NC: Control group, bacterial challenge under normal conditions; HS: Experimental group, hypoxia stress and bacterial challenge.

The clean reads were assembled into 99,774 transcripts with a total length of 7,752,922 nucleotides. The average length of a transcript was 768.27 bp and the N50 length (the transcript length where the cumulative length of longer transcripts is 50% of the total) was 1,414. The size-distribution of these unigenes is shown in Fig. S1.

### Functional annotation and classification

Unigenes annotated using the BLAST^43^ algorithm against the Nr, Swiss-Prot, GO, COG and KEGG^44–48^ databases were illustrated by a Venn diagram in Fig. S2 (Fig. S2 cited from the earlier publication of Zhang *et al*.^42^, the data of sequencing and De novo assembly used in this paper and article of Zhang *et al*. were from the same sequencing project. Therefore, the De novo assembly result is the same, but RNA-seq data for expression analysis are different).

Classification of the 99,774 transcripts in GO functions using the Blast2GO software is illustrated in Fig. S3 (Fig. S3 cited from the earlier publication of Zhang *et al*.^42^). The biological process category had 21 subcategories with cellular processes and metabolic processes as the major ones. The category of cellular component contained 16 subcategories with cell and cell part as the dominant ones. The molecular function contained 12 subcategories with binding and catalytic activity having the largest number of unigenes.

The comparison of different treatment groups revealed that the largest number of differentially expressed genes (DEGs) was at 24 h between the blank group and the normal condition (Table 2). The GO functional classifications of the DEGs at each sampling time are shown in Additional file 2: Fig. S4A–E.

**Table 2:** Counts of the number of DEGs based on comparisons between treatment groups at each sampling time.

Pair	Cellular component (CC)	Molecular function (MF)	Biological process (BP)	All DEGs
BC-4h-VS-NC-4h Up-regulated	1684	1839	3849	7372
BC-4h-VS-NC-4h Down-regulated	628	607	1049	2284
BC-12h-VS-NC-12h Up-regulated	338	443	955	1736
BC-12h-VS-NC-12h Down-regulated	838	1016	1800	3654
BC-24h-VS-NC-24h Up-regulated	1910	2317	5035	9262
BC-24h-VS-NC-24h Down-regulated	1112	698	1375	3185
BC-48h-VS-NC-48h Up-regulated	729	724	1330	2783
BC-48h-VS-NC-48h Down-regulated	483	515	1072	2070
NC-0h-VS-HS-0h Up-regulated	2035	2480	5130	9645
NC-0h-VS-HS-0h Down-regulated	1569	857	1678	4104
NC-4h-VS-HS-4h Up-regulated	619	749	1431	2799
NC-4h-VS-HS-4h Down-regulated	913	659	1357	2929
NC-12h-VS-HS-12h Up-regulated	1853	1991	3995	7839
NC-12h-VS-HS-12h Down-regulated	564	536	882	1982
NC-24h-VS-HS-24h Up-regulated	1714	1851	3445	7010
NC-24h-VS-HS-24h Down-regulated	1017	779	1561	3357
NC-48h-VS-HS-48h Up-regulated	637	551	1077	2265
NC-48h-VS-HS-48h Down-regulated	2730	2303	4356	9389

Sampling time including NC/BC, HS/NC at 4 h, 12 h, 24 h and 48 h. The comparison of BC and NC (NC/BC) at 24 h produced most DEGs (12,447). BC: Blank control group, normal conditions; NC: Control group, bacterial challenge under normal conditions; HS: Experimental group, hypoxia stress and bacterial challenge.

**Figure 1:**
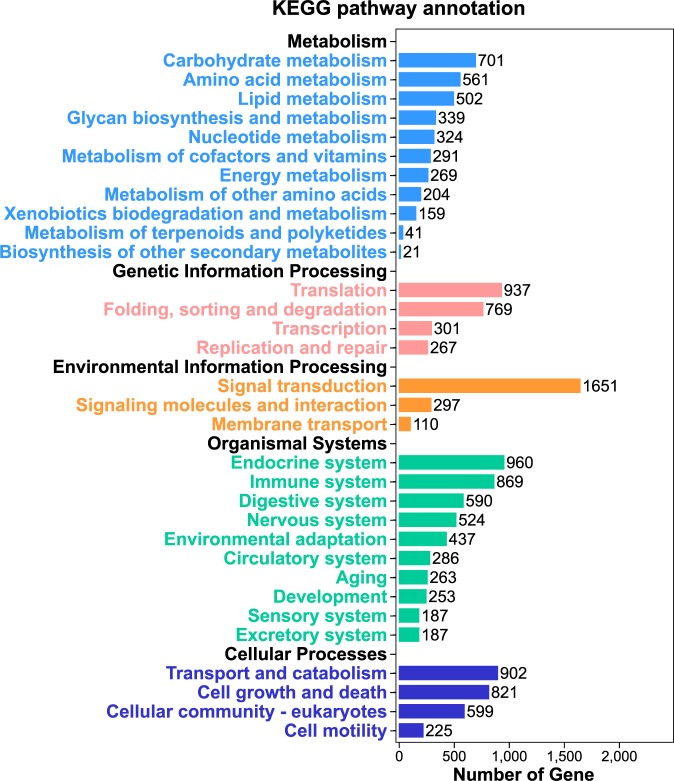
The Kyoto Encyclopedia of Genes and Genomes (KEGG) classification of all the assembled unigenes. A total of 10,840 unigenes had significant matches in the KEGG database and were assigned to five KEGG categories.

The number of 10,840 unigenes were assigned significant matches to the KEGG database. A number were assigned to the pathways of immune system and environmental adaptation. The gene function enrichment analysis (Fig. 1) indicated that 225 unigenes were annotated with immunologic function and mapped into several immune-related pathways, including the MAPK, P38, NF-κB, HIF, and PI3K-AKT signaling pathway (Additional file 1: Table S2)

### Analysis of expression patterns of immune - related genes

The expression of 225 immune-related genes revealed that the expressions of 90 unigenes were related to the PI3K-AKT signal pathway, 62 unigenes were related to the MAPK signal pathway, 20 were related to the NF-κB signal pathway, 24 to the P53 signal pathway, 18 to the HIF signal pathway, 7 to the Toll-like receptor signaling pathway and 4 unigenes were heat shock responsive.

Further, The RPKM relative expression in the control group (NC) was compared with the blank group (BC) and the fold change between groups (NC/BC) was calculated to show the change of gene expression as a result of the infection by the bacteria. The expression significantly increased at 4 h after the infection, but there was a significant decrease at 24 h and 48 h after infection (Additional file 2: Fig. S5). The RPKM relative expression between groups (HS/NC) was calculated. Most of these genes showed a significant decrease in expression after 4 h and 48 h of infection, only a few gene expressions were found to be up-regulated at 12 h and 24 h (Additional file 2: Fig. S6).

### Analysis of DEGs and trends of gene expression among different samples

**Figure 2:**
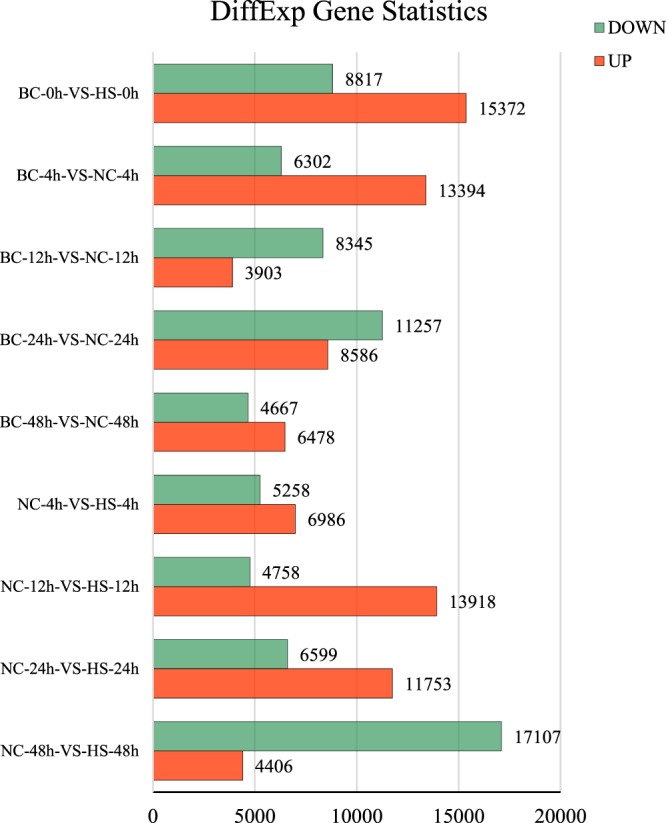
The number of DEGs between different groups at all sample times (4 h, 12 h, 24 h and 48 h after injection). BC: Blank control group, normal conditions; NC: Control group, bacterial challenge under normal conditions; HS: Experimental group, hypoxia stress and bacterial challenge. There were 24189 DEGs between BC-0h and HS-0h (HS-0h/BC-0h) and this number of DEGs accounted for 24% of all unigenes, the largest number of DEGs in all groups. Second, there were 21513 DEGs between NC-48h and HS-48h (HS-48h/NC-48h), accounting for 21.5% of all unigenes. FDR and log2FC were used to screen the DEGs, and the screening condition was FDR < 0.05 and |log2FC| > 1 by edgeR (An R software package for processing significant differences between paired samples).

Between BC-0h and HS-0h (HS-0h/BC-0h), there were 24189 DEGs (24% of all unigenes) identified, the largest number in all group comparisons. Between NC-48h and HS-48h (HS-48h/NC-48h), there were 21,513 DEGs accounting for 21.5% of all unigenes. The detailed numbers of DEGs among each pair of samples were shown in Fig. 2.

Trend analyses performed using the software STEM (Short Time-series Expression Miner) indicated that three trend analyses were designed to track changes over time after the different treatments Trend analysis I (BC-0h, BC-4h, BC-12h, BC-24h, BC-48h), trend analysis II (BC-0h, NC-4h, NC-12h, NC-48h), and Trend analysis III (HS-0h, HS-4h, HS-12h, HS-24h, HS-48h). A considerable number of the genes were found progressively down-regulated over time after *V*. *parahaemolyticus* infection. Also, with time duration after bacterium infection after hypoxia stress (trend analysis III), a total of 11,813 unigenes became down-regulated, accounting for 48% of the total DEGs (24,246) (Additional file 2: Fig. S7).

### Validation of the RNA-seq data by qRT-PCR

Most of the 225 immune-related genes were significantly differentially expressed after exposure to hypoxia stress and bacterial challenge (HS/NC). The validation of the DEGs revealed by RNA-Seq data with qRT-PCR analysis confirmed the expression profiles of *H*. *diversicolor* in response to hypoxia and bacterial infection (Additional file 1: Table S3).

The expression of each gene in the RPKM pathway in the experimental group (HS) was compared with the control group (NC) and the fold change between groups (HS/NC) was calculated to show the change of gene expression as a result of the hypoxia plus bacterial infection. In addition, the relative expression in the control group (NC) was compared with the blank group (BC) and the NC/BC fold change was calculated to show the change as a result of infection by bacteria alone. The relative expression of 24 of the 41 genes (*P38*, *RAC1*, *ASK1*, *MAPKAPK2*, *TRAF2*, *PPP5C*, *MAX*, *MEF2C*, *PRAK*, *bax*, *Apaf1*, *caspas3*, *caspas9*, *caspas10*, *caspas6*, *P53*, *MYD88*, *NF-κB*, *PI3K*, *EIF4B*, *EIF4E*, *FAK*, *IKK*, *AKT*) was lower after hypoxia plus bacterial infection (HS) than with bacterial infection alone (NC) at the early stage (4 h). However, the expression of these genes increased at 12 h and 24 h and then decreased at 48 h after hypoxia plus bacterial infection (HS). After bacterial infection alone, the expression of these genes increased rapidly at 4 h and then declined over time when compared with blank control (BC) (Additional file 2: Fig. S8).

Analysis of qRT-PCR (Additional file 2: Fig. S9) showed that 27 of the differential expressed unigenes were consistent with the results of the transcriptome analysis with the same expression pattern. A higher expression under hypoxic stress at 12 h and 24 h, and a significantly lower relative expression level at 48 h post infection. These genes were found to be mainly associated with signaling pathways involved in immunity, including: the PI3K-AKT signaling pathway (*AKT*, *PI3K*, *EIF4B*, *FAK*, *IKK*, *FASLG*, *ILK*), the MAPK signaling pathway (*MSK1*, *MKKK5*, *NLK*, *MKK3*, *P38*), and the P53 signaling pathway (*P53*, *caspas3*, *caspas8*, *caspas6*).

**Figure 3:**
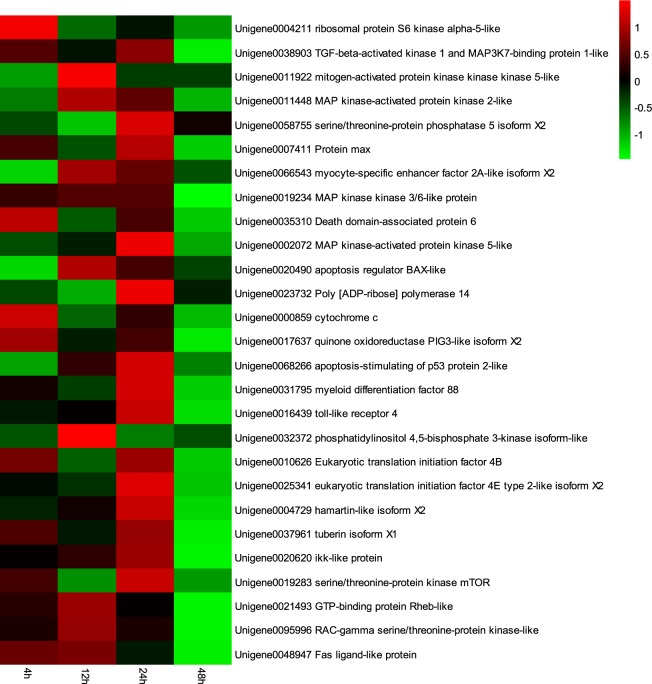
A heat map of the RPKM values for the relative expression of 27 immune-related genes under hypoxia stress and bacterial challenge (Experimental group, HS). The color scale at the far right of the heat map represents the RPKM relative expression value (log2 HS/NC), where red, green and black colors indicate up-regulation, down-regulation and unaltered expression, respectively, relative to the NC Control group.

**Figure 4:**
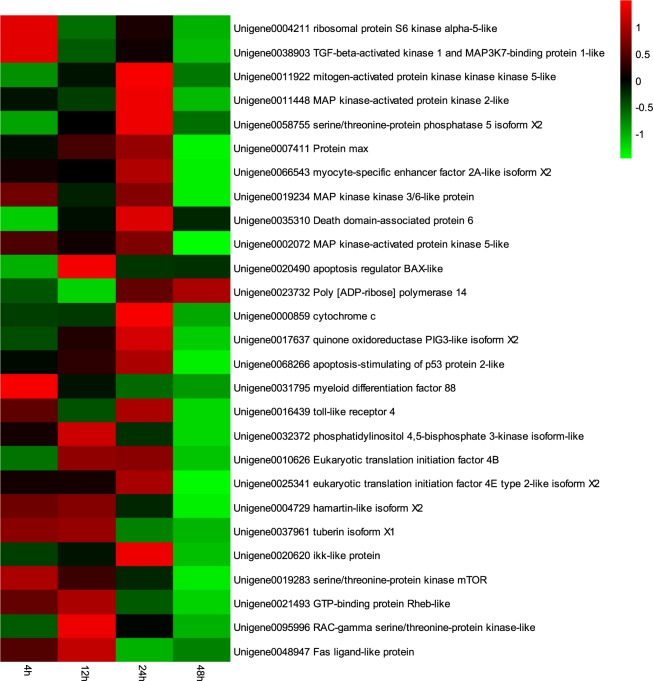
A heat map of the relative expression using qRT-PCR of 27 immune-related genes at hypoxia stress and bacterial challenge (Experimental group, HS). The color scale at the far right of the heat map represents the relative mRNA expression level (log2 HS/NC), where red, green and black colors indicate up-regulation, down-regulation and unaltered expression, respectively, relative to the NC Control group.

The log2 (fold change) of the RPKM values for each group was calculated, and a heat map was used to exhibit the expression of the 41 immune-related genes. All 27 immune-related genes had the same expression pattern. The RPKM analysis of these 41 immune-related genes (Fig. 3) and the qRT-PCR analysis (Fig. 4) showed that the data from qRT-PCR were consistent with the RNA-seq results. The complete heat map of 41 immune-related genes as shown in Additional file 2: Fig. S10.

### Expression of PI3K-AKT signaling pathway-related genes when the *HdAKT* was inhibited by dsRNA

**Figure 5:**
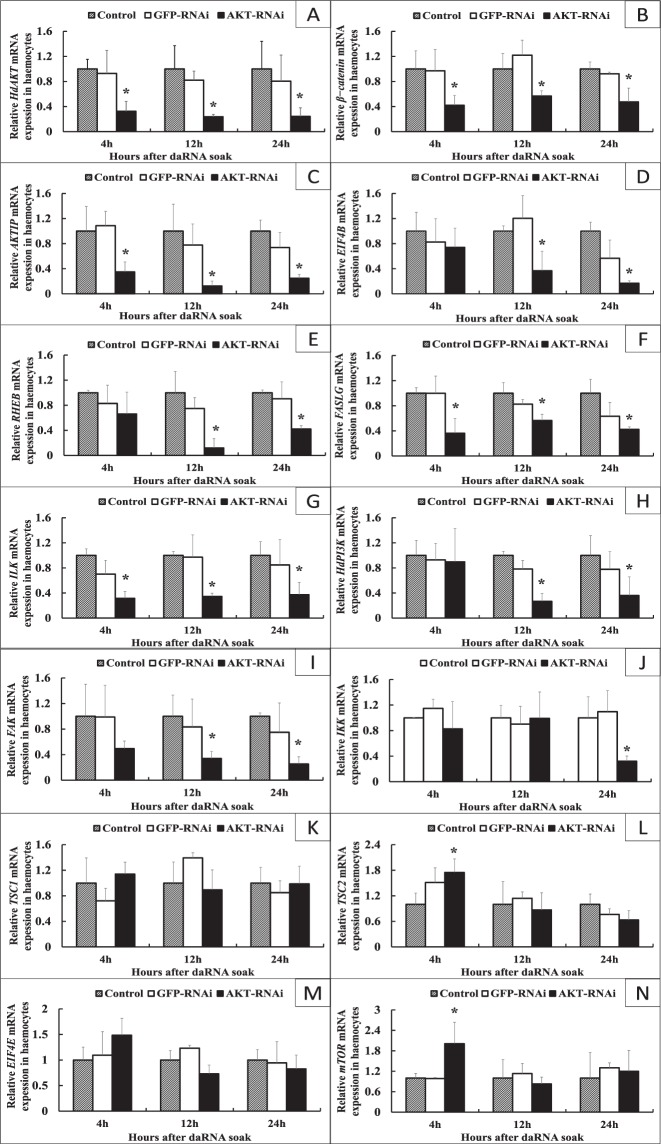
Expression analysis of the PI3K-AKT signaling pathway related genes when the *HdAKT* was inhibited by dsRNA in haemocytes. X axis: Sample times - hours after *HdAKT* was inhibited by dsRNA (4 h, 12 h and 24 h). Y axis: mRNA expression level of the PI3K-AKT signaling pathway related genes. The significant difference between the AKT-RNAi group and the control group is indicated by a (*) at *p* < 0.05. *β-actin* served as reference gene. Control: blank control group. GFP-RNAi: group in which green fluorescent protein (GFP) gene was inhibited by dsRNA. AKT-RNAi: group in which *HdAKT* was inhibited by dsRNA.

The expression of *HdAKT* measured by qRT-PCR showed significantly lower at all times tested (4 h, 12 h, 24 h) than that in the green fluorescent protein (GFP) gene group and the blank control (*p* < 0.05) (Fig. 5A).

After *HdAKT* was inhibited by dsRNA in hemocytes, other genes in the PI3K-AKT signaling pathway showed that the expression of *AKTIP*, *ß−catenin*, *HdAKT*, *FALSG*, and *ILK* were significantly lower at 4 h, expression of *AKTIP*, *ß−catenin*, *HdPI3K*, *EIF4B*, *FAK*, *RHEB*, *HdAKT*, *FALSG* and *ILK* were significantly lower at 12 h, and expression of *AKTIP*, *ß−catenin*, *HdPI3K*, *EIF4B*, *FAK*, *IKK*, *RHEB*, *HdAKT*, *FALSG* and *ILK* were significantly lower at 24 h compared with the GFP group and the blank control group (Fig. 5B–J).

**Figure 6:**
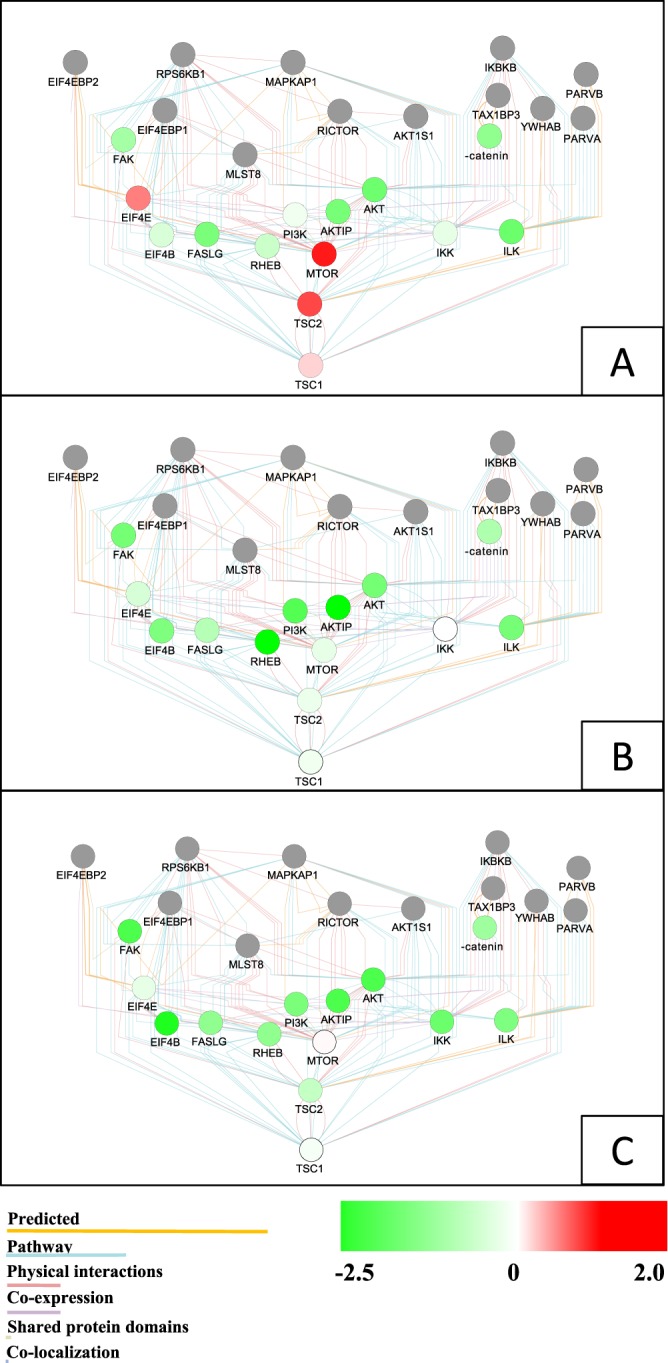
The networks of the PI3K-AKT signaling pathway related genes, representing the level of regulation of PI3K-AKT signaling pathway related genes compared with the blank control group in haemocytes, at various times when the *HdAKT* was inhibited by dsRNA. (**A**) after 4 h, (**B**) after 12 h, (**C**) after 24 h. Red, green and white colors indicate up-regulation, down-regulation and unaltered expression, respectively. The genes not subject to research are marked in gray.

The expressions of *TSC1* and *EIF4E* indicated no significant effect (*P* > 0.05) in all phases after the *HdAKT* was inhibited by dsRNA. But in contrast, the expressions of *TSC2* and *mTOR* were significantly higher compared with that in the GFP group and the blank control group after 4 h of *HdAKT* inhibition (*p* < 0.05) (Fig. 5K–N). Overall, most PI3K-AKT signaling pathway-related genes were down-regulated when the *HdAKT* was inhibited by dsRNA, some genes showed up-regulated mRNA expression or no significant changes (*P* > 0.05) in hemocytes. The molecular network of the PI3K-AKT signal pathway genes after *HdAKT* was inhibited was mapped with Cytoscape software (Fig. 6).

## Discussion

Heavy mortality in hot summers and subsequent outbreak of bacterial pathogen occurred often in the farmed *H*. *diversicolor* (“small abalone”) and threatened the sustainability of the economically important industry in China. Stress is well known to suppress immune responses, and investigations into the stress response mechanisms of *H*. *diversicolor* have hypothesized a link between environmental stresses (hypoxia and/or thermal stress) and increased susceptibility to bacterial pathogens^10,11,13,14^. One of the vital factors that hinders the management of disease outbreaks is the inadequacy of our understanding of the molecular basis of the abalone’s environmental stress response. Thus, studying the de novo transcriptomic analysis of *H*. *diversicolor* that has been challenged with multiple stressors is of great importance in understanding the molecular mechanisms that suppress the immune response to pathogenic bacteria.

Sequence similarity searches showed that one could assign 48.3% of the unigenes using one or more of the 4 databases used. Of these, most (97.8%) were assigned to the 687 species in the Nr database, which provides a strong foundation for excavating immune-related genes and signaling pathways in the stress response. In the Nr database, 17% of unigenes showed homology with *Pacific oysters*, and 15% of unigenes showed homology with the gastropod *Elysia chlorotica*. Most of the top-ranked species were marine mollusks. These results indicate that the transcript data has high reliability.

Stress levels are usually the result of many interactions among multiple environmental variables and their intensity, duration and frequency. In the present study, non-redundant genes from the Nr and Swissprot databases indicated a large number of DEGs of *H*. *diversicolor* were involved in environmental stress and/or bacterial infection. This kind of change in gene expression levels upon stress and bacterial challenge is common, and it has been observed in previous work in our lab^10^.

### Analysis of GO and KEGG annotation

As expected, the annotation of the unigenes by Blast2GO identified genes involved in a variety of cellular activities. 22.6% of the unigenes were allocated to functional categories. This allows us to quickly find the target genes and locate the relevant signaling pathways.

Annotation of unigenes in the KEGG pathway database showed that many pathways may be related to the immune defense response of *H*. *diversicolor*. Multiple immune-related and apoptosis-related pathways were found, including the MAPK signaling pathway, the P38 pathway, the NF-κB signaling pathway, the HIF signaling pathway under hypoxia stress, heat shock genes that play an important role in high temperature stress, and the PI3K-AKT pathway with immunosuppression under stress. The discovery and identification of these immune-related pathways has laid a foundation for the study of the mechanism of immune function genes under various environmental stressors.

### Differentially expressed genes and qRT-PCR validation

The number of DEGs in each group varies, with different expression patterns among the groups. Not surprisingly, the largest number of DEGs was between the blank control and the hypoxia stressed group, immediately after the injection. 24% of all unigenes were differentially expressed in this comparison, where, 59% of these genes were annotated indicates that the DEGs can provide very useful information about the responses to bacterial challenge and environmental stress. In addition, the results of qRT-PCR analyses of those genes showed that the expression patterns over time were in agreement with the Illuminia RNA-Seq analyses.

### Discovery of immune-related genes

Of the large number of unigenes identified through RNAseq, a relatively small number were related to immune function. As described in the introduction, the PI3K-AKT signal transduction pathway is an evolutionarily conserved innate immune pathway, which plays a critical role to resist external pathogens and stressors from invertebrates to vertebrates^49–53^. One report on *Crassostrea hongkongensis*^54^ suggested that AKT1 participates vigorously in the immune response against microbial pathogens and heat shock stresses. Additionally, PI3K signaling pathway was reported to involve in regulating glycogen metabolism in *Pinctada fucata*^55^. Furthermore, transcriptome analysis showed that PI3K-AKT signaling pathway involved in immune defense response to pathogenic infection and environmental stress in *Mytilus coruscus*^56^, *Onchidium struma*^57^ and *Meretrix petechialis*^58^. In addition, the PI3K-AKT signaling pathway related to TLR4-mediated immune defense^59,60^, that similar with the abalone *H*. *diversicolor* after the *V*. *parahaemolyticus* stimulation^14^.

The expression of *AKT*, *PI3K*, *EIF4B*, *FAK*, *IKK*, *FALSG*, *ILK* and other related genes in the PI3K-AKT signal pathway under hypoxia stress decreased compared to those under normal conditions as the time after bacterial infection increased, and at 48 h after infection no differences were found between the hypoxia stress group and control group. However, the expression of PI3K-AKT signaling pathway-related genes increased rapidly at 4 h after bacterial infection and then declined over time compared with blank control (NC/BC), suggesting that this pathway is involved in the rapid immune response to infection. Also, hypoxia stress delays this rapid immune response, indicating that the PI3K-AKT signaling pathway may be involved in hypoxia-induced immunosuppression of *H*. *diversicolor* under hypoxia stress when infected with *V*. *parahaemolyticus*. Therefore, further research on the PI3K-AKT signaling pathway under the combined environmental stresses and pathogen invasion would be fruitful.

In vertebrates, members of the MAPK signaling pathway belong to the family of protein kinases that catalyze the phosphorylation of proteins and form intricate regulatory networks that regulate gene expression and play a critical functional role in cell proliferation, apoptosis, immune defense and humoral immunity^61^. As important members of the serine/threonine protein kinase family, a cascade reaction of kinases activates nuclear transcription factors in stages through MAPK, MKK and MKKK. This cascade reaction of kinases plays an important role in immune defense and developmental regulation.

The MAPK pathway includes four sub-pathways: an extra-cellular signal-regulated kinase (ERK) signaling pathway, a Jun N-terminal kinase (JNK) pathway, the P38 signaling pathway and an extracellular signal-regulated kinase signaling pathway. These four pathways are interlinked and interact with each other to form an extensive information transmission network that constitutes the regulation mechanism for immune defense and developmental and reproductive function. The MAPK signaling pathway is an important immune-related pathway in vertebrates, and also is involved in immune defense against pathogenic bacteria in mollusks in *Mytilus galloprovincialis*^62^, *Crassostrea ariakensis*^63^ and *Haliotis tuberculate*^64^. Previous transcriptome analysis showed that MAPK signaling pathway involved in immune response, and the expression of these genes varied significantly during bacterial infection, such as *M*. *coruscus*, *O*. *struma*, *M*. *petechialis*, *Paphia undulata* and *Mytilus edulis*^42,56,57,65,66^. Additionally, it is also involved in immune defense under environmental stress in *O*. *struma*^53^. Relatively, little research on this pathway in mollusks has been reported, and the available reports mostly focused onto bacterial immune defense.

In this study, a total of 62 MAPK signaling pathway related genes were obtained, including key factors in multiple pathways: *MSK1*, *MKKK5*, *NLK*, *MKK3*, *P38* etc. Our transcriptome analysis of these related genes in the MAPK signaling pathway showed that the expression levels of these genes appeared to decline to varying degrees after *V*. *parahaemolyticus* infection under hypoxia stress especially at 4 h and 48 h (HS). But the expression levels of these genes increase rapidly at 4 h after bacterial infection and then decline over time compared with blank control. As for the P13K-AKT pathway, this suggests that the MAPK signaling pathway may be involved in the rapid immune response after *V*. *parahaemolyticus* infection and hypoxia-induced immunosuppression of *H*. *diversicolor*. This provides a basis for studying the immune regulation mechanism of the MAPK signaling pathway in more complex environmental stress and pathogen infection experiments.

As a highly conserved pathway from insects to mammals, the nuclear factor-κB (NF-κB), which plays central roles in many important physiological and pathological processes, has been studied extensively in the innate immune system^67,68^. NF-κB is present in most vertebrates and invertebrates; and it has been described as a nuclear factor which can specifically bind to the κB sequence of immunoglobulin κ enhancer^69^. More recently, it has been identified in almost all animal cell types and has become well known as a transcription factor involved in many biological processes such as development, immune defense, inflammatory responses, apoptosis, homeostatic mechanisms and cellular differentiation^70^.

Previous transcriptome analysis have shown that NF-κB signaling pathway plays a pivotal role in immune response, and the expression of these genes varied significantly during bacterial infection in *O*. *struma*, *Concholepas concholepas* and *M*. *edulis*^57,66,71^. Further, in a recent study of the transcriptome KEGG analyses suggested that the NF-κB signaling pathway were actively expressed in the organ and might serve as a host defense modulator against exogenous infections such as bacteria and other pathogens in *Saxidomus purpuratus*^72^. Meanwhile, after recognition of the invasive pathogens, NF-κB signaling pathway are triggered, and this pathway is considered a crucial component in innate immunity, as it plays an important role in the innate defense against common pathogens in *P*. *undulata*^65^. Our previous results have shown that the expressions of NF-κB signaling pathway-related genes were up-regulated significantly under hypoxia stress, thermal stress and both stressors together, as well as bacterial infection^10^. This indicates that the NF-κB signaling pathway is involved in both the innate immune response and in immune regulation under multiple environmental stressors.

In this study, a total of twenty NF-κB signaling pathway related genes were obtained, which provides a foundation for subsequent screening of key genes for immunity. In the transcriptome trend analysis, the expression of *NF-κB* showed a downward trend. But after bacterial infection alone (NC) there was a rapid increase of expression at 4 h. As hypoxia stress delays the rapid immune response after bacterial infection, these results indicate that the NF-κB signaling pathway is involved in immunosuppression of *H*. *diversicolor* under the combined stress of hypoxia stress and vibrio infection.

The P53 signaling pathway plays a key role in the process of apoptosis regulation and is closely related to the biological regulation of cell growth, differentiation and immunity in vertebrates^73^. Our transcriptome analysis revealed twenty-four P53 signaling pathway-related genes that are closely related to apoptosis and immune function in *H*. *diversicolor*. We have also obtained 18 HIF signaling pathway-related genes that are important for the response to a hypoxic environment. In addition, 4 heat shock response-related genes were obtained; these genes are of great significance to cope with a high temperature stimulus.

The 41 immune-related unigenes that were selected on the basis of their roles of genes in various immune pathways as immune-related genes, mainly belonged to the PI3K-AKT, MAPK, NF-κB and P53 signaling pathways. These unigenes were verified by qRT-PCR and the expression patterns of 27 of these unigenes were consistent with the transcriptome analysis.

Based on the analysis of the heat-map and the gene expression patterns, 24 genes in haemocytes that respond to bacterial infection (NC) and hypoxia plus bacterial infection (HS) can be generally classified as showing the following response pattern: The genes increase rapidly at 4 h after bacterial infection alone, and then decline over time compared with blank control (BC), suggesting that they are involved in the rapid immune response to bacterial infection. But after hypoxia plus bacterial infection (HS) they were expressed at a low level compared with bacterial infection (NC) at 4 h, and their expression increased at 12 h and 24 h, then decreased at 48 h. The expression of these genes is thus correlated with the delay in the response to bacterial infection under hypoxia stress. To some extent, these immune-related genes may be involved in the process of hypoxia-induced immunosuppression of *H*. *diversicolor* after *V*. *parahaemolyticus* infection with hypoxia stress. Many of these genes are associated with immune signaling pathways, such as the P13K-AKT pathway.

### The regulation mechanism of PI3K-AKT signaling pathway when the *HdAKT* was inhibited by dsRNA

The technology of gene silencing by dsRNA (RNAi) has been applied recently to marine mollusks, such as a freshwater snail *Biomphalaria glabrata* for studying the function of genes in host-parasite interactions with trematode parasites^74^. The genes activated by AKT indicated the inflammatory or immune response, a cell survival response or cellular proliferation. To study the mechanism of AKT in the PI3K-AKT signaling pathway, the *HdAKT* gene was inhibited by dsRNA in the blood cells of *H*. *diversicolor*.

qRT-PCR showed that the expression of *HdAKT* in the experimental groups inhibited by dsRNA was significantly lower than in the GFP gene group and the blank control, and *HdPI3K* and pathway-related genes were significantly down-regulated in the experimental group of hemocytes. This shows that *HdAKT* plays a positive role in the regulation of the PI3K-AKT signaling pathway. The expression levels of other genes in the P13K-AKT pathway, except *EIF4E*, *TSC1*, *TSC2* and *mTOR*, were lower than that in the control group after *HdAKT* was inhibited for 4 h, indicating *HdAKT* plays a key role in the regulation of PI3K-AKT signaling pathway.

The unchanged expressions of *EIF4E*, *TSC1*, *TSC2* and *mTOR* genes at 4 h and longer after *HdAKT* was inhibited suggested that these genes are located upstream of the PI3K-AKT signaling pathway, and may uni-directionally regulate *HdAKT*. The interactions between PI3K-AKT signaling pathway-related genes were also visualized by Cytoscape after the inhibition of *HdAKT*. From the molecular network, it is reflected that inhibition of *HdAKT* deepens as the inhibition time lengthens, and these genes gradually appear to be inhibited. As a supplementary experiment, the inhibition of *HdAKT* helped to understand the mechanism of PI3K-AKT signaling. These results provide evidence of the PI3K-AKT signaling linkages between these genes in abalone.

## Conclusions

Transcriptome profile of *H*. *diversicolor* hemocytes revealed that the expression of immune defense genes in the PI3K-AKT, MAPK, NF-κB and P53 signaling pathways under hypoxia stress decreased relative to their expression under normal conditions, as the time after bacterial infection increased. But the expression levels of these genes increased rapidly at 4 h after bacterial infection and then declined over time compared with blank control. The results indicated that the hypoxia stress delays this rapid immune response and the immune defense genes may be involved in hypoxia-induced immunosuppression of *H*. *diversicolor*. The silencing of *HdAKT* downregulated the expression of other genes in the P13K-AKT pathway, except *EIF4E*, *TSC1*, *TSC2* and *mTOR*. Furthermore, the gene interaction network of the PI3K-AKT signal pathway was successfully constructed after *HdAKT* was inhibited, by Cytoscape software. These results contribute substantially to exploring the changes of the immune system in marine mollusks under environment stress conditions.

## Materials and Methods

### Animals and sample preparation

Adult small abalone at body length of 6.00 ± 0.50 cm and body weight of 16.45 ± 2.50 g were purchased from Peiyang abalone farm (Xiamen, Fujian Province) in August 2015. Following the methods in our previous publication^10^, these abalone were kept in recirculating systems with sand-filtered seawater at a consistent temperature (25 °C) and dissolved oxygen (DO) (6.2 mg/L), and fed with *Laminaria japonica* once a day. During experimental period, the temperature and the level of DO of the seawater were monitored continuously and maintained using a temperature and dissolved oxygen control system (Xiamen Water Bay Automation Technology Co., Ltd, Xiamen, China). Before the challenge experiments, abalones were acclimated in the system for 10 days.

### Challenge experimental designation

**Figure 7:**
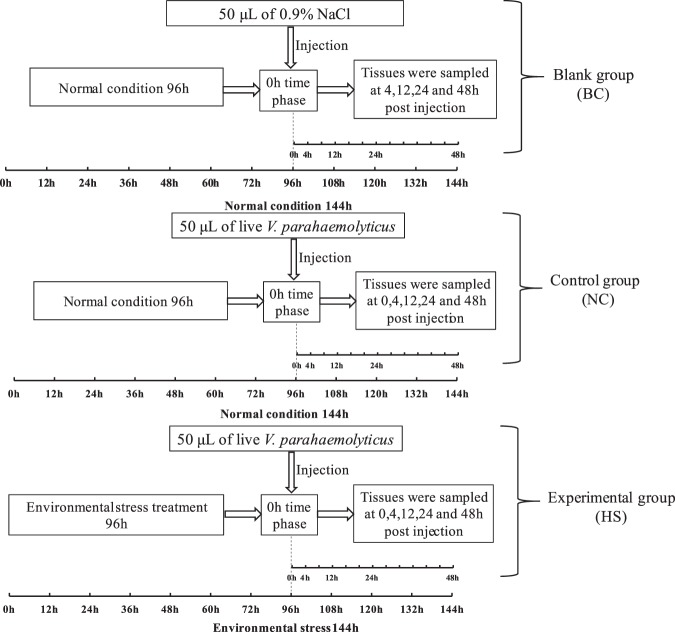
Schematics of the experimental set-up. The Blank group (BC) were kept under normal conditions and injected with 50 μL 0.9% NaCl at 96 h. The Experimental group (HS) were challenged by injection with 50 μL live *V*. *parahaemolyticus* (isolated from diseased abalone) in 0.9% NaCl (1.1 × 10^8^ cfu/mL). These abalone were exposed to the stress of hypoxia during the whole experiment. The Control group (NC) was kept under normal conditions but challenged by injection of 50 μL live *V*. *parahaemolyticus* (isolated from diseased abalone) in 0.9% NaCl (1.1 × 10^8^ cfu/mL) as for HS.

After acclimation, abalones were randomly divided into three groups for the following treatments: (1) Normal control group (NC) subjected to bacterial challenge under normal conditions. After these abalone had been cultured under the normal conditions (T = 25 °C and DO = 6.2 mg/L) for 96 h, all individuals were injected with 50 μL live *V*. *parahaemolyticus* in 0.9% NaCl (1.1 × 10^8^ cfu/mL) into the foot muscle. (2) Blank control group (BC) with no bacterial challenge under normal conditions. After these abalone had been cultured under normal conditions for 96 h, they were injected with 50 μL of 0.9% NaCl. (3) An experimental group subjected to bacterial challenge after hypoxia stress (HS). These abalone were cultured at 25 °C and DO of 2 mg/L for 96 h, and then injected with 50-μL of live *V*. *parahaemolyticus* in 0.9% NaCl (1.1 × 10^8^ cfu/mL) into the foot muscle. After the treatments of injection stated above, the abalone were returned to their original tanks containing seawater at T = 25 °C and DO = 6.2 mg/L. At 0, 4, 12, 24 and 48 h post-injection, the abalones from each treatment group were sampled and tissues were collected as follows: Hemolymph was drew through cutting off the foot using a syringe with 21-gauge needle, and centrifuged at 2000 × g at 4 °C for 10 min to separate hemocytes (He). The hemocytes were immediately stored in liquid nitrogen until used for RNA isolation (Fig. 7). As 0 h was sampled immediately after injection, BC-0h is equivalent to NC-0h, and only the BC group was sampled. In all of the treatments, at least six abalone were sampled at each time.

### Double-stranded RNA (dsRNA) generation and exposure assay

A fragment of *HdAKT* (whose complete cDNA has been cloned with GenBank accession No. KX056492) and green fluorescent protein (GFP) gene from the pEGFP-N1 vector were amplified by PCR using gene-specific primers (Additional file 1: Table S4). The primers of the *HdAKT* fragment include sense primers and antisense primers of both *HdAKT* and *gfp* genes for transcribing single-stranded RNA). The PCR products were purified, sequenced and transcribed into single-stranded RNA (ssRNA) using the T7 phage RNA polymerases (Promega, Madison, WI, USA), and then the DNA template was degraded by DNase I (Promega) at a ratio of 1 U/μg. The sense ssRNA and antisense ssRNA were mixed and annealed at 75 °C for 15 min, 65 °C for 15 min, and then cool down to room temperature (T = 25 °C) at the rate of 0.1 °C/s. The quality of dsRNA and size shift was assessed by agarose (1%) gel electrophoresis and the concentration of the dsRNA was assessed by spectrophotometry (NanoDrop ND-1000, Thermo Fisher Scientific, Wilmington, DE, USA).

To achieve the RNA interference (RNAi), the exogenous dsRNA at a final concentration of 5 μg/ml was added directly to the hemocyte culture medium without any vehicle. The *HdAKT* dsRNA was applied to hemocytes in the experimental group, the GFP dsRNA was applied to hemocytes in the control group (GFP group), and medium without RNAi treatment was paralleled as blank control. After mixing and incubation at 27 °C for 6, 12 and 24 h, the hemocytes were collected by centrifugation 2000 × g and stored in liquid nitrogen until they were processed to isolate the RNA and detect the mRNA expression by qRT-PCR. Six replicates from each treatment group were produced by using different individual abalones.

### Isolation of total RNA and RNA-sequencing

Total RNA was extracted from all the hemocyte samples using the E.Z.N.A.® Total RNA Kit II (Omega Bio-tek, Inc.Norcross, GA, USA) following the manufacturer’s instructions. The mRNA quality was assessed by agarose (1%) gel electrophoresis and quantified by spectrophotometry (NanoDrop ND-1000). RNA sequencing was then performed by Gene Denovo Biotechnology Co. (Guangzhou, China). As input material, 3-μg RNA per sample was used for sequencing libraries and the RNA was enriched by Oligo (dT) beads. Afterwards, the enriched mRNA was cut into short fragments and reverse transcribed into fragmented cDNA with random primers. Then, the fragments of cDNA were purified with a QiaQuick PCR extraction kit (QIAGEN China Co., Ltd., Shanghai, China), end repaired, poly(A) primer was added, and the DNA ligated to Illumina sequencing adapters. Finally, the ligation products were selected by agarose gel electrophoresis and amplified by PCR. The cDNA libraries of all samples were sequenced using the Illumina HiSeq 2000 platform (Illumina, San Diego, CA, USA) following the manufacturer’s recommendations, and paired-end reads were generated.

For qRT-PCR, the total mRNA was reverse transcribed into cDNA with 1-µg total RNA and 2-µL of 10-µM random primers by M-MLV reverse transcriptase (Promega -Beijing Biotech Co., Ltd., Beijing, China). The cDNA was diluted 100-fold and stored at -20 °C, ready for qRT-PCR.

### *De novo* assembly, functional annotation, and identification of DEGs

Trinity software was used to assemble the clean reads as described for *De Novo* transcriptome assembly without a reference genome, from which adaptor sequences, ambiguous ‘N’ nucleotides (with a ratio of ‘N’ more than 10%) and low quality sequences (with quality score <5) were removed^75^. The raw sequence data of fourteen libraries have been deposited into the NCBI Sequence Read Archive (SRA) under the accession number SRP166258. For annotation analysis, non-redundant sequences were subjected to public databases, including NCBI (http://www.ncbi.nlm.nih.gov/), non-redundant protein (Nr) and non-redundant nucleotide (Nt), Swiss-Prot (http://www.ebi.ac.uk/uniprot/), Gene Ontology (GO) (http://www.geneontology.org/), Clusters of Orthologous Groups (COG) (http://www.ncbi.nlm.nih.gov/COG/) and Kyoto Encyclopedia of Genes and Genomes (KEGG) (http://www.genome.jp/kegg/). When the results of different databases were conflicted, a priority order of alignments from Nr, Nt, KEGG, Swiss-Prot, GO to the COG databases was followed.

All clean sequencing reads of the fourteen libraries were mapped to the transcriptome assembly using the software Bowtie2 with the default setting. Gene expression levels of unigenes were based on read counts obtained by RNA Sequence Expected Maximization, and these read counts were normalized using the Reads per Kilobase per Million mapped transformation^76,77^. However, differentially expressed genes (DEGs) were further analyzed by the “Identifying edge” R package (www.r-project.org) to assign them to genes in cDNA libraries of *H*. *diversicolor*. In the edge R statistics, files (FDR < 0.05 and |log2FC| > 1) were set as the threshold for significantly differential expression to identify DEGs in various libraries.

### qRT-PCR verification

To further investigate the roles of genes in immune pathways, 41 representative genes were screened and selected to validate the RNA-Seq data by qRT-PCR. Gene-specific primers of these 41 genes (Additional file 1: Table S5) were designed to amplify products of 200–300 bp of the cDNA. A 10 × SYBR Green Master mix (Promega) was used for qRT-PCR according to the manufacturer’s protocol, and the *β-actin* gene (Accession No. AY436644) was selected as the housekeeping gene due to its stable expression in abalones^8,11,12^.

qRT-PCR was carried out in a Light Cycler 480 Roche Real-time Thermal Cycler (Roche, Switzerland) with a 20-µL reaction volume containing 9 µL of 1:100 diluted original cDNA, 10 µL of 10 × SYBR Green Master Mix (Promega), and 0.5 µL of each primer (10 µM). The cycling conditions for the PCR reaction were as follows: 1 min at 95 °C, followed by 40 cycles at 95 °C for 15 s, 60 °C for 1 min. Melting curves were also plotted (60 °C - 90 °C) to make sure that a single PCR product was amplified for each pair of primers. The comparative CT method (ΔCT = CT of target gene minus CT of *β-actin* gene and ΔΔCT = ΔCT of any sample minus calibrator sample) was used to calculate the relative expression level of all these genes. The t-test by IBM SPSS Statistics 20 was used to determine the difference in the mean values among the treatments, with a significance level of *p* < 0.05. A heat map was created using the R Programming Language (version 3.4.0) to visualize the gene expression data. The gene networks of signaling pathway related genes were constructed using the Gene MANIA Cytoscape app (Cytoscape 3.4.0).

## Supplementary information

Supplementary information 1

Supplementary information 2
