# Supplementary material for: Immunity-related genes and signaling pathways under hypoxic stresses in Haliotis diversicolor: a transcriptome analysis
Source: Sci Rep. 2019 Dec 24;9:19741. doi: 10.1038/s41598-019-56150-2 (PMC6930256; doi:10.1038/s41598-019-56150-2)
Supplement: Supplementary file 1 — Supplementary information 1 [file 41598_2019_56150_MOESM1_ESM.docx]

**Additional file 1:**

**Immunity genes and signaling pathways under hypoxic stresses in *Haliotis diversicolor*: a transcriptome analysis**

Yulong Sun^1,3^, Xin Zhang^1,3^, Yilei Wang^2^*, Robert Day^4^, Huiping Yang^5^, and Ziping Zhang^1,2^*

1 College of Animal Science, Fujian Agriculture and Forestry University, Fuzhou 350002, PR China

2 Fisheries College, Jimei University, Xiamen, 361021, China

3 Key Laboratory of Marine Biotechnology of Fujian Province, Institute of Oceanology, Fujian Agriculture and Forestry University, Fuzhou 350002, PR China

4 School of Biosciences, University of Melbourne, Parkville, Victoria, Australia 3010

5 School of Forest Resources and Conservation, IFAS, University of Florida, 7922 NW 71st Street, Gainesville, FL 32615

Table S1 The acronyms used in this study

| Acronyms | Gene name |
| --- | --- |
| HdAKT | serine/threonine-protein kinase |
| CASP3 | cysteinyl aspartate specific proteinase 3 |
| CASP6 | cysteinyl aspartate specific proteinase 6 |
| CASP7 | cysteinyl aspartate specific proteinase 7 |
| CASP8 | cysteinyl aspartate specific proteinase 6 |
| CASP9 | cysteinyl aspartate specific proteinase 9 |
| CASP10 | cysteinyl aspartate specific proteinase 10 |
| DAXX | death-associated protein 6 |
| EIF4B | translation initiation factor 4B |
| EIF4E | translation initiation factor 4E |
| FAK | focal adhesion kinase |
| FASLG | tumor necrosis factor ligand superfamily member 6 |
| IKK | inhibitor of nuclear factor kappa-b kinase |
| ILK | integrin-linked protein kinase |
| MKK3 | mitogen-activated protein kinase kinase 3 |
| MKKK5 | mitogen-activated protein kinase kinase kinase 5 |
| mTOR | mammalian Target of Rapamycin |
| MYD88 | myeloid differentiation factor 88 |
| NF-κB | nuclear factor κB |
| NLK | nemo like kinase |
| P38 | p38 kinase |
| HdPI3K | phosphatidylinositol-4,5-bisphosphate 3-kinase |
| RAC1 | Ras-related C3 botulinum toxin substrate 1 |
| RHEB | Ras homolog enriched in brain |
| TAB1 | TAK1-binding protein 1 |
| Toll4 | toll-like receptor 4 |
| TRAF2 | TNF receptor-associated factor 2 |
| TSC1 | tuberous sclerosis complex 1 |
| TSC2 | tuberous sclerosis complex 2 |
| β-catenin | beta-catenin |

Table S2 225 unigenes of immune-related pathways

| **Unigene-ID** | | **Gene name** | **Matching species** | **length** | **E value** | **ident**（%） | **Nr-Score** |  |
| --- | --- | --- | --- | --- | --- | --- | --- | --- |
| **PI3K-AKT signaling pathway** | | |  |  |  |  |  |  |
| Unigene0068986 | | PREDICTED: glycogen [starch] synthase-like | *Aplysia californica* | 1607 | 0 | 82 | 957 |  |
| Unigene0095996 | | PREDICTED: RAC-gamma serine/threonine-protein kinase-like | *Aplysia californica* | 2126 | 0 | 77 | 751 |  |
| Unigene0032372 | | PREDICTED: phosphatidylinositol 4,5-bisphosphate 3-kinase catalytic subunit beta isoform-like | *Aplysia californica* | 2303 | 0 | 60 | 925 |  |
| Unigene0047660 | | PREDICTED: glucose-6-phosphatase 2-like isoform X2 | *Aplysia californica* | 1209 | 9E-83 | 50 | 265 |  |
| Unigene0072997 | | PREDICTED: phosphatidylinositol 3,4,5-trisphosphate 3-phosphatase and dual-specificity protein phosphatase PTEN-like | *Crassostrea gigas* | 1148 | 1E-139 | 70 | 416 |  |
| Unigene0006078 | | PREDICTED: phosphoenolpyruvate carboxykinase, cytosolic [GTP]-like | *Aplysia californica* | 3257 | 0 | 76 | 1053 |  |
| Unigene0016704 | | PREDICTED: cyclin-dependent kinase 6-like | *Crassostrea gigas* | 5429 | 5E-160 | 72 | 501 |  |
| Unigene0030696 | | PREDICTED: bcl-2-like protein 2 | *Aplysia californica* | 2798 | 1E-68 | 58 | 235 |  |
| Unigene0031752 | | PREDICTED: cyclin-dependent kinase 2-like | *Aplysia californica* | 1708 | 1E-169 | 79 | 493 |  |
| Unigene0005332 | | PREDICTED: phosphatidylinositol 3-kinase regulatory subunit alpha-like | *Aplysia californica* | 5014 | 0 | 60 | 1225 |  |
| Unigene0048228 | | calcium-dependent protein kinase C | *Aplysia californica* | 3919 | 0 | 80 | 1077 |  |
| Unigene0011586 | | 40S ribosomal protein S6 | *Aplysia californica* | 1499 | 6E-100 | 87 | 310 |  |
|  | |  |  |  |  |  |  |  |
|  | |  |  |  |  |  |  |  |
| **Unigene-ID** | | **Gene name** | **Matching species** | **length** | **E value** | **ident**（%） | **Nr-Score** |  |
| Unigene0001991 | | PREDICTED: glycogen synthase kinase-3 beta-like | *Crassostrea gigas* | 6792 | 0 | 81 | 691 |  |
| Unigene0013979 | | PREDICTED: son of sevenless homolog 2-like | *Crassostrea gigas* | 6411 | 0 | 60 | 1426 |  |
| Unigene0010626 | | Eukaryotic translation initiation factor 4B | *Crassostrea gigas* | 5270 | 3E-57 | 64 | 224 |  |
| Unigene0025341 | | PREDICTED: eukaryotic translation initiation factor 4E type 2-like isoform X2 | *Crassostrea gigas* | 2231 | 3E-118 | 83 | 363 |  |
| Unigene0001954 | | PREDICTED: serine/threonine-protein phosphatase 2A 65 kDa regulatory subunit A alpha isoform-like isoform X2 | *Crassostrea gigas* | 3094 | 0 | 89 | 1077 |  |
| Unigene0095295 | | SCO-spondin | *Crassostrea gigas* | 323 | 2E-06 | 32 | 52.4 |  |
| Unigene0012552 | | PREDICTED: serine/threonine-protein phosphatase 2A 55 kDa regulatory subunit B alpha isoform-like | *Aplysia californica* | 5780 | 0 | 86 | 859 |  |
| Unigene0005180 | | Deleted in malignant brain tumors 1 protein | *Crassostrea gigas* | 322 | 8E-19 | 41 | 89 |  |
| Unigene0055229 | | PREDICTED: fibroblast growth factor 8-like | *Aplysia californica* | 695 | 1E-16 | 38 | 82.4 |  |
| Unigene0061531 | | PREDICTED: epidermal growth factor receptor-like | *Aplysia californica* | 1193 | 1E-58 | 50 | 201 |  |
| Unigene0084085 | | fibroblast growth factor receptor 2-like | *Pomacea canaliculata* | 269 | 2.00E-32 | 60 | 124 |  |
|  | |  |  |  |  |  |  |  |
|  | |  |  |  |  |  |  |  |
| **Unigene-ID** | | **Gene name** | **Matching species** | **length** | **E value** | **ident**（%） | **Nr-Score** |  |
| Unigene0028561 | | PREDICTED: growth factor receptor-bound protein 2-like isoform X1 | *Crassostrea gigas* | 7611 | 7E-84 | 60 | 284 |  |
| Unigene0023895 | | PREDICTED: dual specificity mitogen-activated protein kinase kinase 1-like isoform X1 | *Aplysia californica* | 3979 | 0 | 81 | 654 |  |
| Unigene0031843 | | PREDICTED: mitogen-activated protein kinase 1 | *Aplysia californica* | 3838 | 0 | 88 | 684 |  |
| Unigene0012884 | | ApCREB2 protein | *Aplysia californica* | 1764 | 1E-28 | 38 | 124 |  |
| Unigene0048277 | | serine/threonine-protein phosphatase 2A catalytic subunit beta isoform isoform X1 | Crassostrea gigas | 2065 | 0 | 97 | 642 |  |
| Unigene0048947 | | Fas ligand-like protein | *Haliotis discus discus* | 1124 | 6E-175 | 74 | 499 |  |
| Unigene0032860 | | Ras-related C3 botulinum toxin substrate 1, partial | *Pomacea canaliculata* | 3715 | 2.00E-114 | 95 | 936 |  |
| Unigene0022793 | | PREDICTED: caspase-2-like | *Crassostrea gigas* | 2946 | 7E-66 | 34 | 235 |  |
| Unigene0022768 | | PREDICTED: tyrosine-protein kinase JAK2-like | *Crassostrea gigas* | 6185 | 6E-114 | 28 | 402 |  |
| Unigene0069209 | | PREDICTED: cyclic AMP-dependent transcription factor ATF-2-like | *Crassostrea gigas* | 744 | 6E-43 | 61 | 160 |  |
| Unigene0020620 | | ikk-like protein | *Pinctada fucata* | 6360 | 0 | 59 | 900 |  |
| Unigene0022820 | | insulin-related peptide receptor | *Pinctada fucata* | 9102 | 0 | 49 | 1350 |  |
| Unigene0015297 | | G protein B subunit | *Crassostrea angulata* | 4573 | 0 | 96 | 681 |  |
|  | |  |  |  |  |  |  |  |
|  | |  |  |  |  |  |  |  |
| **Unigene-ID** | | **Gene name** | **Matching species** | **length** | **E value** | **ident**（%） | **Nr-Score** |  |
| Unigene0079570 | | PREDICTED: guanine nucleotide-binding protein subunit beta-5-like | *Aplysia californica* | 759 | 9E-85 | 88 | 266 |  |
| Unigene0005739 | | PREDICTED: thrombospondin-1-like | *Aplysia californica* | 2975 | 2E-38 | 41 | 151 |  |
| Unigene0019621 | | PREDICTED: ribosomal protein S6 kinase beta-1-like | *Crassostrea gigas* | 6111 | 0 | 80 | 803 |  |
| Unigene0043711 | | PREDICTED: tyrosine kinase receptor Cad96Ca | *Lottia gigantea* | 233 | 2.00E-27 | 62 | 110 |  |
| Unigene0059458 | | PREDICTED: fibroblast growth factor receptor 1-like | *Aplysia californica* | 2069 | 1E-83 | 44 | 277 |  |
| Unigene0026722 | | Fibroblast growth factor receptor | *Crassostrea gigas* | 1071 | 2E-83 | 49 | 274 |  |
| Unigene0065452 | | PREDICTED: vascular endothelial growth factor receptor 3-like | *Aplysia californica* | 1061 | 1E-179 | 75 | 54030273 |  |
| Unigene0020215 | | PREDICTED: hepatocyte growth factor receptor-like | *Aplysia californica* | 4754 | 0 | 44 | 840 |  |
| Unigene0020329 | | Fibroblast growth factor receptor | *Crassostrea gigas* | 3064 | 2E-87 | 46 | 302 |  |
| Unigene0059615 | | Tenascin-X | *Crassostrea gigas* | 300 | 3E-12 | 40 | 69.3 |  |
| Unigene0026369 | | fibrinogen-related protein 8 | *Mytilus galloprovincialis* | 1215 | 1E-25 | 56 | 111 |  |
| Unigene0015207 | | PREDICTED: laminin subunit gamma-1-like | *Crassostrea gigas* | 8572 | 0 | 59 | 1989 |  |
| Unigene0030707 | | Sushi domain-containing protein 2 | *Crassostrea gigas* | 4721 | 1E-06 | 27 | 60.5 |  |
| Unigene0020205 | | PREDICTED: integrin beta-1-like isoform X1 | *Aplysia californica* | 4501 | 0 | 65 | 1000 |  |
| Unigene0024099 | | PREDICTED: focal adhesion kinase 1-like isoform X3 | *Crassostrea gigas* | 6975 | 0 | 62 | 1403 |  |
| Unigene0029856 | | PREDICTED: tyrosine-protein kinase SYK-like | *Crassostrea gigas* | 5372 | 0 | 57 | 725 |  |
|  | |  |  |  |  |  |  |  |
|  | |  |  |  |  |  |  |  |
| **Unigene-ID** | | **Gene name** | **Matching species** | **length** | **E value** | **ident**（%） | **Nr-Score** |  |
| Unigene0022180 | | PREDICTED: serine/threonine-protein kinase N2-like | *Aplysia californica* | 5111 | 0 | 66 | 1303 |  |
| Unigene0038855 | | collagen pro alpha-chain | *Haliotis discus* | 4461 | 0 | 90 | 1759 |  |
| Unigene0010127 | | PREDICTED: collagen alpha-2(IV) chain-like | *Crassostrea gigas* | 6638 | 0 | 57 | 1367 |  |
| Unigene0012276 | | PREDICTED: laminin subunit alpha-like | *Crassostrea gigas* | 7058 | 0 | 42 | 1226 |  |
| Unigene0064450 | | Multiple epidermal growth factor-like domains 9 | *Crassostrea gigas* | 501 | 4E-24 | 37 | 103 |  |
| Unigene0064210 | | PREDICTED: laminin subunit beta-1-like | *Aplysia californica* | 321 | 2E-48 | 69 | 174 |  |
| Unigene0031512 | | PREDICTED: reelin-like isoform X1 | *Crassostrea gigas* | 2850 | 0 | 51 | 734 |  |
| Unigene0024438 | | PREDICTED: ryncolin-4-like | *Crassostrea gigas* | 2493 | 4E-54 | 44 | 196 |  |
| Unigene0006981 | | 3-phosphoinositide-dependent protein kinase 1-like isoform X1 | *Crassostrea gigas* | 2718 | 0 | 72 | 686 |  |
| Unigene0086051 | | PREDICTED: integrin alpha-4-like | *Aplysia californica* | 302 | 3E-14 | 36 | 75.5 |  |
| Unigene0058823 | | PREDICTED: integrin alpha-PS1-like isoform X2 | *Aplysia californica* | 276 | 4E-13 | 53 | 71.6 |  |
| Unigene0098581 | | PREDICTED: integrin alpha-PS1-like isoform X2 | *Aplysia californica* | 247 | 1E-26 | 65 | 110 |  |
| Unigene0011669 | | PREDICTED: integrin alpha-8-like | *Aplysia californica* | 7695 | 0 | 55 | 1145 |  |
| Unigene0047784 | | hypothetical protein LOTGIDRAFT_238363 | *Lottia gigantea* | 2283 | 5E-13 | 0.48 | 75.9 |  |
| Unigene0021432 | | PREDICTED: G1/S-specific cyclin-E-like isoform X2 | *Crassostrea gigas* | 2939 | 0 | 62 | 546 |  |
| Unigene0020880 | | 14-3-3 zeta | *Haliotis diversicolor* | 4179 | 0 | 100 | 564 |  |
|  | |  |  |  |  |  |  |  |
|  | |  |  |  |  |  |  |  |
| **Unigene-ID** | | **Gene name** | **Matching species** | **length** | **E value** | **ident**（%） | **Nr-Score** |  |
| Unigene0022758 | | 5'-AMP-activated protein kinase catalytic subunit alpha 2 | *Cellana toreuma* | 6094 | 0 | 83 | 874 |  |
| Unigene0019283 | | serine/threonine-protein kinase mTOR | *Pinctada martensii* | 9901 | 0 | 78 | 3982 |  |
| Unigene0020691 | | PREDICTED: regulatory-associated protein of mTOR-like, partial | *Crassostrea gigas* | 3664 | 0 | 55 | 678 |  |
| Unigene0009832 | | PREDICTED: eukaryotic translation initiation factor 4E-binding protein 1-like | *Crassostrea gigas* | 3363 | 1E-35 | 64 | 140 |  |
| Unigene0004728 | | PREDICTED: hamartin-like | *Aplysia californica* | 1693 | 1E-128 | 45 | 416 |  |
| Unigene0037961 | | PREDICTED: tuberin isoform X1 | *Mizuhopecten yessoensis* | 4655 | 0 | 52 | 1330 |  |
| Unigene0021493 | | PREDICTED: GTP-binding protein Rheb-like | *Crassostrea gigas* | 1504 | 2E-88 | 74 | 278 |  |
| Unigene0018419 | | PREDICTED: optineurin-like | *Crassostrea gigas* | 2941 | 2E-92 | 36 | 317 |  |
| Unigene0060023 | | PREDICTED: serine/threonine-protein kinase STK11 isoform X1 | *Lottia gigantea* | 2799 | 1.00E-177 | 72 | 530 |  |
| Unigene0017811 | | GTP binding protein Ras | *Haliotis discus discus* | 4165 | 4E-117 | 99 | 372 |  |
| Unigene0032478 | | lethal with SEC13 protein 8 | *Pinctada martensii* | 2704 | 0 | 82 | 553 |  |
| Unigene0062794 | | retinoid X receptor | *Haliotis diversicolor* | 2081 | 0 | 99 | 904 |  |
| Unigene0000749 | | PREDICTED: cyclic AMP-dependent transcription factor ATF-2-like | *Crassostrea gigas* | 952 | 2E-32 | 57 | 133 |  |
|  | |  |  |  |  |  |  |  |
|  | |  |  |  |  |  |  |  |
| **Unigene-ID** | | **Gene name** | **Matching species** | **length** | **E value** | **ident**（%） | **Nr-Score** |  |
| Unigene0079777 | | PREDICTED: cyclic AMP-responsive element-binding protein 3-like protein 2 isoform X1 | *Crassostrea gigas* | 3166 | 5E-146 | 61 | 457 |  |
| Unigene0070024 | | PREDICTED: cyclic AMP-dependent transcription factor ATF-6 beta-like | *Crassostrea gigas* | 1140 | 1E-78 | 41 | 260 |  |
| Unigene0014886 | | PREDICTED: myb-related protein B-like | *Aplysia californica* | 2573 | 3E-53 | 58 | 204 |  |
| Unigene0006095 | | glucose-regulated protein 94 | *Crassostrea gigas* | 2930 | 0 | 79 | 1185 |  |
| Unigene0013122 | | PREDICTED: hsp90 co-chaperone Cdc37-like | *Aplysia californica* | 1895 | 1E-150 | 68 | 449 |  |
| Unigene0070402 | | PREDICTED: G1/S-specific cyclin-D2-like | *Crassostrea gigas* | 3161 | 6E-112 | 66 | 357 |  |
| Unigene0016439 | | PREDICTED: toll-like receptor 4 | *Aplysia californica* | 4037 | 3E-60 | 26 | 231 |  |
| Unigene0027766 | | toll-like receptor m | *Mytilus galloprovincialis* | 4473 | 4E-113 | 31 | 386 |  |
| Unigene0021115 | | PREDICTED: integrin-linked protein kinase-like isoform X2 | *Crassostrea gigas* | 5009 | 0 | 83 | 806 |  |
| **MAPK signaling pathway** | | |  |  |  |  |  |  |
| Unigene0027926 | | PREDICTED: caspase-7-like | *Pomacea canaliculata* | 1716 | 2.00E-64 | 43 | 223 |  |
| Unigene0035310 | | Death domain-associated protein 6 | *Crassostrea gigas* | 3209 | 2E-49 | 43 | 198 |  |
| Unigene0090282 | | Tumor necrosis factor ligand superfamily member 14 | *Crassostrea gigas* | 1279 | 8E-07 | 31 | 55.5 |  |
| Unigene0057926 | | PREDICTED: TNF receptor-associated factor 2-like | *Crassostrea gigas* | 2788 | 4E-177 | 47 | 533 |  |
| Unigene0028796 | | TRAF6 | *Pinctada martensii* | 5905 | 3E-173 | 48 | 550 |  |
| Unigene0021453 | | heat shock cognate protein 70 | *Haliotis diversicolor* | 2699 | 0 | 99 | 621 |  |
|  | |  |  |  |  |  |  |  |
|  | |  |  |  |  |  |  |  |
| **Unigene-ID** | | **Gene name** | **Matching species** | **length** | **E value** | **ident**（%） | **Nr-Score** |  |
| Unigene0009999 | | Eka-protein kinase A protein | *Lingula anatina* | 6994 | 4.00E-103 | 82 | 346 |  |
| Unigene0054594 | | PREDICTED: guanine nucleotide-binding protein subunit alpha-13-like | *Aplysia californica* | 7042 | 0 | 78 | 583 |  |
| Unigene0017211 | | calcineurin A | *Haliotis discus discus* | 4340 | 0 | 99 | 1008 |  |
| Unigene0099109 | | Ras GTPase-activating protein 1 | *Crassostrea gigas* | 516 | 2E-73 | 66 | 232 |  |
| Unigene0045518 | | PREDICTED: ragulator complex protein LAMTOR3-A-like | *Aplysia californica* | 1122 | 5E-42 | 55 | 151 |  |
| Unigene0018546 | | Mnk | *Aplysia californica* | 3654 | 2E-173 | 68 | 325 |  |
| Unigene0013274 | | PREDICTED: ribosomal protein S6 kinase 2 beta-like | *Aplysia californica* | 5035 | 0 | 85 | 1295 |  |
| Unigene0038596 | | PREDICTED: serum response factor-like | *Aplysia californica* | 2082 | 1E-102 | 47 | 332 |  |
| Unigene0090563 | | transcription factor fos-like 2 | *Mytilus galloprovincialis* | 2236 | 3E-22 | 37 | 103 |  |
| Unigene0012028 | | PREDICTED: inner centromere protein-like isoform X1 | *Crassostrea gigas* | 2484 | 6E-97 | 60 | 312 |  |
| Unigene0083573 | | PREDICTED: growth arrest and DNA damage-inducible protein GADD45 gamma-like | *Aplysia californica* | 1426 | 2E-35 | 42 | 137 |  |
| Unigene0038903 | | PREDICTED: TGF-beta-activated kinase 1 and MAP3K7-binding protein 1-like | *Crassostrea gigas* | 2047 | 0 | 57 | 597 |  |
| Unigene0006923 | | PREDICTED: TGF-beta-activated kinase 1 and MAP3K7-binding protein 3-like isoform X2 | *Crassostrea gigas* | 3049 | 7E-45 | 47 | 180 |  |
|  | |  |  |  |  |  |  |  |
|  | |  |  |  |  |  |  |  |
| **Unigene-ID** | | **Gene name** | **Matching species** | **length** | **E value** | **ident**（%） | **Nr-Score** |  |
| Unigene0005678 | | PREDICTED: evolutionarily conserved signaling intermediate in Toll pathway, mitochondrial-like | *Aplysia californica* | 1306 | 1E-121 | 55 | 371 |  |
| Unigene0031040 | | Serine/threonine-protein kinase mig-15 | *Pomacea canaliculata* | 3930 | 0 | 77 | 1575 |  |
| Unigene0021122 | | PREDICTED: serine/threonine-protein kinase PAK 3-like isoform X1 | *Crassostrea gigas* | 5668 | 0 | 82 | 845 |  |
| Unigene0069161 | | PREDICTED: mitogen-activated protein kinase kinase kinase 1-like isoform X3 | *Aplysia californica* | 3649 | 0 | 59 | 965 |  |
| Unigene0022104 | | PREDICTED: mitogen-activated protein kinase kinase kinase 2-like isoform X1 | *Crassostrea gigas* | 5930 | 8E-178 | 52 | 563 |  |
| Unigene0024854 | | PREDICTED: serine/threonine-protein kinase 26-like, partial | *Crassostrea gigas* | 4170 | 6E-139 | 43 | 448 |  |
| Unigene0076326 | | PREDICTED: mitogen-activated protein kinase kinase kinase MLT-like, partial | *Aplysia californica* | 269 | 3E-41 | 74 | 148 |  |
| Unigene0011922 | | PREDICTED: mitogen-activated protein kinase kinase kinase 5-like | *Aplysia californica* | 3196 | 0 | 69 | 1326 |  |
| Unigene0030679 | | PREDICTED: mitogen-activated protein kinase kinase kinase 7-like isoform X5 | *Aplysia californica* | 7079 | 0 | 56 | 734 |  |
| Unigene0043175 | | MAPKKK4 mitogen activated protein kinase kinase kinase 4 | *Ostrea edulis* | 285 | 2E-43 | 75 | 149 |  |
|  | |  |  |  |  |  |  |  |
|  | |  |  |  |  |  |  |  |
| **Unigene-ID** | | **Gene name** | **Matching species** | **length** | **E value** | **ident**（%） | **Nr-Score** |  |
| Unigene0017554 | | PREDICTED: serine/threonine-protein kinase TAO1-like isoform X1 | *Aplysia californica* | 4380 | 0 | 67 | 1216 |  |
| Unigene0050556 | | PREDICTED: dual specificity mitogen-activated protein kinase kinase 4-like isoform X3 | *Aplysia californica* | 744 | 6E-84 | 77 | 265 |  |
| Unigene0078821 | | PREDICTED: dual specificity mitogen-activated protein kinase kinase 7-like | *Aplysia californica* | 2400 | 0 | 68 | 581 |  |
| Unigene0003417 | | PREDICTED: dual specificity mitogen-activated protein kinase kinase 6-like | *Aplysia californica* | 4931 | 5E-159 | 72 | 497 |  |
| Unigene0098174 | | PREDICTED: rho guanine nucleotide exchange factor 10-like protein | *Crassostrea gigas* | 224 | 2E-06 | 40 | 51.6 |  |
| Unigene0018416 | | PREDICTED: filamin-B-like | *Aplysia californica* | 5932 | 0 | 61 | 1540 |  |
| Unigene0017908 | | PREDICTED: adapter molecule Crk-like | *Crassostrea gigas* | 4493 | 7E-105 | 65 | 342 |  |
| Unigene0016113 | | PREDICTED: beta-arrestin-1-like | *Aplysia californica* | 4676 | 0 | 92 | 759 |  |
| Unigene0021757 | | c-Jun N-terminal kinase JNK | *Aplysia californica* | 7614 | 0 | 85 | 714 |  |
| Unigene0028387 | | PREDICTED: mitogen-activated protein kinase 14A-like isoform X2 | *Crassostrea gigas* | 6042 | 0 | 77 | 570 |  |
| Unigene0002072 | | PREDICTED: MAP kinase-activated protein kinase 5-like | *Crassostrea gigas* | 4469 | 5E-144 | 61 | 458 |  |
| Unigene0011448 | | PREDICTED: MAP kinase-activated protein kinase 2-like | *Aplysia californica* | 2750 | 0 | 83 | 618 |  |
|  | |  |  |  |  |  |  |  |
|  | |  |  |  |  |  |  |  |
| **Unigene-ID** | | **Gene name** | **Matching species** | **length** | **E value** | **ident**（%） | **Nr-Score** |  |
| Unigene0011970 | | PREDICTED: ribosomal protein S6 kinase alpha-5-like | *Aplysia californica* | 545 | 8E-104 | 87 | 328 |  |
| Unigene0050046 | | Nuclear factor of activated T-cells 5 | *Crassostrea gigas* | 576 | 5E-73 | 63 | 246 |  |
| Unigene0070921 | | transcription factor activator protein-1 | *Haliotis diversicolor* | 2827 | 0 | 100 | 645 |  |
| Unigene0007411 | | Protein max | *Crassostrea gigas* | 1458 | 2.00E-51 | 66 | 182 |  |
| Unigene0066543 | | PREDICTED: myocyte-specific enhancer factor 2A-like isoform X2 | *Aplysia californica* | 4687 | 3E-124 | 56 | 407 |  |
| Unigene0036035 | | PREDICTED: mucin-19-like | *Aplysia californica* | 7468 | 2E-115 | 67 | 414 |  |
| Unigene0058755 | | PREDICTED: serine/threonine-protein phosphatase 5 isoform X2 | *Crassostrea gigas* | 1506 | 0 | 76 | 671 |  |
| Unigene0068776 | | PREDICTED: protein phosphatase 1B-like | *Aplysia californica* | 4123 | 0 | 76 | 621 |  |
| Unigene0015695 | | PREDICTED: dual specificity mitogen-activated protein kinase kinase 5-like | *Crassostrea gigas* | 3031 | 3E-157 | 51 | 481 |  |
| Unigene0088978 | | PREDICTED: mitogen-activated protein kinase 7-like | *Aplysia californica* | 4318 | 0 | 61 | 725 |  |
| Unigene0012444 | | Mitogen-activated protein kinase kinase kinase 2 | *Crassostrea gigas* | 5225 | 8E-84 | 29 | 309 |  |
| Unigene0008684 | | Serine/threonine protein kinase NLK | *Crassostrea gigas* | 6205 | 0 | 86 | 757 |  |
| Unigene0072512 | | calcium channel beta subunit splice isoform A- | *Lymnaea stagnalis* | 2751 | 0 | 73 | 843 |  |
| Unigene0014567 | | tyrosine /threonine phosphatase | *Patella vulgata* | 529 | 3E-39 | 68 | 142 |  |
|  | |  |  |  |  |  |  |  |
|  | |  |  |  |  |  |  |  |
| **Unigene-ID** | | **Gene name** | **Matching species** | **length** | **E value** | **ident**（%） | **Nr-Score** |  |
| Unigene0019439 | | calcineurin B | *Haliotis discus discus* | 3546 | 3E-105 | 99 | 337 |  |
| Unigene0018047 | | PREDICTED: ras-related protein R-Ras2-like | *Aplysia californica* | 3390 | 2E-95 | 81 | 311 |  |
| Unigene0031750 | | PREDICTED: ras-related protein M-Ras-like | *Pomacea canaliculata* | 5047 | 3.00E-89 | 89 | 299 |  |
| Unigene0046906 | | PREDICTED: ras-like protein 3 isoform X2 | *Crassostrea gigas* | 2877 | 9E-104 | 87 | 330 |  |
| Unigene0020516 | | PREDICTED: rap guanine nucleotide exchange factor 2-like isoform X4 | *Aplysia californica* | 10035 | 0 | 65 | 1500 |  |
| Unigene0060073 | | PREDICTED: neurofibromin-like isoform X10 | *Crassostrea gigas* | 2696 | 0 | 72 | 984 |  |
| Unigene0029516 | | PREDICTED: ras guanyl-releasing protein 3-like | *Aplysia californica* | 5767 | 0 | 76 | 1037 |  |
| **p53 signaling pathway** | | |  |  |  |  |  |  |
| Unigene0068266 | | PREDICTED: apoptosis-stimulating of p53 protein 2-like | *Crassostrea gigas* | 3628 | 0 | 49 | 781 |  |
| Unigene0024976 | | PREDICTED: GRIP and coiled-coil domain-containing protein 1-like | *Aplysia californica* | 2460 | 2E-162 | 45 | 499 |  |
| Unigene0007834 | | cyclin-dependent kinase 1 | *Haliotis diversicolor supertexta* | 1856 | 0 | 99 | 627 |  |
| Unigene0039645 | | PREDICTED: apoptosis regulator BAX-like | *Aplysia californica* | 544 | 3E-77 | 69 | 239 |  |
| Unigene0036915 | | PREDICTED: serine/threonine-protein kinase Chk1-like | *Crassostrea gigas* | 1798 | 0 | 73 | 689 |  |
| Unigene0018850 | | caspase-8 | *Haliotis discus discus* | 2788 | 0 | 74 | 968 |  |
|  | |  |  |  |  |  |  |  |
|  | |  |  |  |  |  |  |  |
| **Unigene-ID** | | **Gene name** | **Matching species** | **length** | **E value** | **ident**（%） | **Nr-Score** |  |
| Unigene0018770 | | PREDICTED: E3 ubiquitin-protein ligase SIAH1-like | *Aplysia californica* | 2446 | 2E-167 | 81 | 495 |  |
| Unigene0062772 | | PREDICTED: serine-protein kinase ATM-like | *Crassostrea gigas* | 1491 | 2E-132 | 66 | 403 |  |
| Unigene0086460 | | cyclin B | *Haliotis diversicolor supertexta* | 2020 | 0 | 99 | 853 |  |
| Unigene0012678 | | PREDICTED: CD82 antigen-like | *Aplysia californica* | 2085 | 2E-35 | 32 | 141 |  |
| Unigene0080174 | | PREDICTED: serine/threonine-protein kinase ATR-like | *Crassostrea gigas* | 1226 | 3E-81 | 41 | 279 |  |
| Unigene0082880 | | PREDICTED: serine/threonine-protein kinase Chk2-like | *Aplysia californica* | 1966 | 0 | 76 | 757 |  |
| Unigene0000859 | | cytochrome c | *Mytilus edulis* | 2035 | 7.00E-54 | 83 | 190 |  |
| Unigene0031591 | | PREDICTED: G2 and S phase-expressed protein 1-like isoform X1 | *Crassostrea gigas* | 3191 | 1E-37 | 30 | 157 |  |
| Unigene0017637 | | PREDICTED: quinone oxidoreductase PIG3-like isoform X2 | *Crassostrea gigas* | 2288 | 8E-88 | 68 | 289 |  |
| Unigene0024300 | | PREDICTED: etoposide-induced protein 2.4 homolog | *Crassostrea gigas* | 2970 | 1E-124 | 56 | 392 |  |
| Unigene0002515 | | PREDICTED: DNA damage-binding protein 2-like | *Crassostrea virginica* | 1675 | 2.00E-146 | 55 | 441 |  |
| Unigene0013800 | | Sestrin-1 | *Crassostrea gigas* | 5625 | 0 | 61 | 699 |  |
| Unigene0090126 | | PREDICTED: metalloreductase STEAP4-like | *Crassostrea gigas* | 2395 | 0 | 58 | 569 |  |
| Unigene0068915 | | PREDICTED: E3 ubiquitin-protein ligase RFWD2-like | *Crassostrea gigas* | 623 | 6E-130 | 87 | 388 |  |
|  | |  |  |  |  |  |  |  |
|  | |  |  |  |  |  |  |  |
| **Unigene-ID** | | **Gene name** | **Matching species** | **length** | **E value** | **ident**（%） | **Nr-Score** |  |
| Unigene0078376 | | PREDICTED: RING finger and CHY zinc finger domain-containing protein 1-like isoform X1 | *Crassostrea gigas* | 1121 | 5E-106 | 61 | 323 |  |
| Unigene0010315 | | PREDICTED: cyclin-G1-like | *Crassostrea gigas* | 3404 | 9E-39 | 37 | 155 |  |
| Unigene0016981 | | Protein phosphatase 1D | *Crassostrea gigas* | 4643 | 9E-137 | 54 | 447 |  |
| Unigene0091379 | | PREDICTED: ribonucleoside-diphosphate reductase small chain-like | *Crassostrea gigas* | 1600 | 0 | 75 | 624 |  |
| **NF-kB signaling pathway** | | |  |  |  |  |  |  |
| Unigene0025594 | | PREDICTED: sperm motility kinase-like | *Crassostrea gigas* | 5293 | 7E-87 | 49 | 308 |  |
| Unigene0085234 | | PREDICTED: nuclear factor NF-kappa-B p105 subunit-like isoform X2 | *Aplysia californica* | 1325 | 3E-162 | 65 | 494 |  |
| Unigene0068031 | | PREDICTED: casein kinase II subunit alpha isoform X2 | *Aplysia californica* | 8106 | 0 | 94 | 664 |  |
| Unigene0007378 | | PREDICTED: casein kinase II subunit beta | *Aplysia californica* | 1508 | 2E-158 | 97 | 459 |  |
| Unigene0097278 | | PREDICTED: TNF receptor-associated factor 3 | *Pomacea canaliculata* | 432 | 9.00E-69 | 80 | 227 |  |
| Unigene0028612 | | PREDICTED: baculoviral IAP repeat-containing protein 7-like | *Crassostrea gigas* | 4395 | 2E-117 | 38 | 392 |  |
| Unigene0031795 | | myeloid differentiation factor 88 | *Haliotis diversicolor* | 1166 | 0 | 97 | 697 |  |
| Unigene0028128 | | IL-1 receptor associated kinase 4 | *Haliotis diversicolor* | 5757 | 0 | 99 | 1068 |  |
| Unigene0023870 | | bactericidal permeability increasing protein | *Crassostrea gigas* | 4748 | 2E-116 | 41 | 384 |  |
| Unigene0028368 | | hypothetical protein CAPTEDRAFT_193665 | *Mizuhopecten yessoensis* | 1588 | 9.00E-09 | 57 | 65.1 |  |
|  | |  |  |  |  |  |  |  |
|  | |  |  |  |  |  |  |  |
| **Unigene-ID** | | **Gene name** | **Matching species** | **length** | **E value** | **ident**（%） | **Nr-Score** |  |
| Unigene0034161 | | Tumor necrosis factor ligand superfamily member 14 | *Crassostrea gigas* | 1962 | 8E-09 | 27 | 62.4 |  |
| Unigene0015768 | | PREDICTED: tyrosine-protein kinase SRK2-like isoform X3 | *Crassostrea gigas* | 4200 | 3E-50 | 84 | 195 |  |
| Unigene0030273 | | phospholipase C gamma | *Aplysia californica* | 6930 | 0 | 38 | 800 |  |
| Unigene0003003 | | PREDICTED: B-cell lymphoma/leukemia 10-like | *Crassostrea gigas* | 1171 | 6E-20 | 36 | 92.8 |  |
| Unigene0059577 | | PREDICTED: mucosa-associated lymphoid tissue lymphoma translocation protein 1-like | *Crassostrea gigas* | 2889 | 0 | 40 | 556 |  |
| Unigene0025570 | | PREDICTED: B-cell linker protein-like | *Aplysia californica* | 5974 | 1E-18 | 39 | 99 |  |
| Unigene0013955 | | PREDICTED: SUMO-conjugating enzyme UBC9-B | *Aplysia californica* | 1417 | 1E-98 | 89 | 302 |  |
| Unigene0017241 | | PREDICTED: tumor necrosis factor alpha-induced protein 3-like isoform X3 | *Crassostrea gigas* | 4110 | 8E-91 | 29 | 325 |  |
| Unigene0059635 | | PREDICTED: prostaglandin G/H synthase 2-like isoform X1 | *Crassostrea gigas* | 3838 | 0 | 66 | 839 |  |
| Unigene0021915 | | PREDICTED: probable ATP-dependent RNA helicase DDX58 isoform X3 | *Crassostrea gigas* | 3399 | 0 | 44 | 578 |  |
| **Toll-like receptor signaling pathway** | | |  |  |  |  |  |  |
|  | |  |  |  |  |  |  |  |
|  | |  |  |  |  |  |  |  |
| **Unigene-ID** | | **Gene name** | **Matching species** | **length** | **E value** | **ident**（%） | **Nr-Score** |  |
| Unigene0024872 | | Fas-associated protein with death domain | *Mytilus galloprovincialis* | 2315 | 2E-21 | 27 | 101 |  |
| Unigene0017461 | | tumor necrosis factor receptor-associated factor 3 | *Pinctada fucata* | 1545 | 2E-45 | 43 | 174 |  |
| Unigene0024156 | | PREDICTED: toll-like receptor 13 | *Crassostrea gigas* | 2600 | 9E-85 | 29 | 294 |  |
| Unigene0021905 | | PREDICTED: toll-interacting protein-like | *Aplysia californica* | 2712 | 5E-115 | 66 | 362 |  |
| Unigene0005368 | | PREDICTED: toll-like receptor 3 | *Aplysia californica* | 2095 | 5E-63 | 34 | 234 |  |
| Unigene0032466 | | serine/threonine-protein kinase TBK1-like | *Aplysia californica* | 4781 | 0 | 47 | 715 |  |
| Unigene0024157 | | PREDICTED: toll-like receptor 13 | *Crassostrea gigas* | 544 | 1E-14 | 31 | 78.6 |  |
| **Heat shock protein related genes** | | |  |  |  |  |  |  |
| Unigene0022032 | | heat shock protein 90 | *Haliotis diversicolor* | 3170 | 0 | 92 | 1396 |  |
| Unigene0058883 | | PREDICTED: hsp70-binding protein 1-like | *Crassostrea gigas* | 2618 | 6E-105 | 51 | 336 |  |
| Unigene0059412 | | PREDICTED: hsp70-Hsp90 organizing protein 1-like | *Crassostrea gigas* | 1159 | 4E-74 | 48 | 239 |  |
| Unigene0003616 | | HSPB1-associated protein 1 homolog isoform X1 | *Crassostrea gigas* | 1636 | 3E-99 | 52 | 318 |  |
| **HIF-1 signaling pathway** | | |  |  |  |  |  |  |
| Unigene0015483 | | hypoxia inducible factor-1alpha | *Haliotis diversicolor* | 4586 | 0 | 95 | 1465 |  |
| Unigene0048182 | | PREDICTED: pyruvate dehydrogenase E1 component subunit beta, mitochondrial-like | *Crassostrea gigas* | 1373 | 0 | 77 | 604 |  |
| Unigene0005873 | PREDICTED: hexokinase-2-like | | *Crassostrea gigas* | 3015 | 0 | 80 | 751 |  |
| Unigene0032374 | | phosphatidylinositol 4,5-bisphosphate 3-kinase catalytic subunit delta isoform-like isoform X1 | *Crassostrea gigas* | 900 | 2E-57 | 39 | 206 |  |
| Unigene0015943 | | 6-phosphofructo-2-kinase/fructose-2,6-bisphosphatase 1-like isoform X7 | *Crassostrea gigas* | 2335 | 0 | 75 | 697 |  |
|  | |  |  |  |  |  |  |  |
|  | |  |  |  |  |  |  |  |
| **Unigene-ID** | | **Gene name** | **Matching species** | **length** | **E value** | **ident**（%） | **Nr-Score** |  |
| Unigene0022358 | | PREDICTED: histone-lysine N-methyltransferase 2D-like | *Aplysia californica* | 5602 | 2E-54 | 54 | 217 |  |
| Unigene0030954 | | RING-box protein | *Haliotis diversicolor supertexta* | 469 | 1E-78 | 99 | 238 |  |
| Unigene0073547 | | PREDICTED: cullin-2-like | *Crassostrea gigas* | 1751 | 0 | 76 | 891 |  |
| Unigene0037092 | | PREDICTED: von Hippel-Lindau disease tumor suppressor-like | *Crassostrea gigas* | 1983 | 2E-16 | 32 | 84.7 |  |
| Unigene0047872 | | Transcription elongation factor B polypeptide 1 | *Crassostrea gigas* | 1475 | 2.00E-75 | 95 | 243 |  |
| Unigene0030278 | | PREDICTED: transcription elongation factor B polypeptide 2-like isoform X2 | *Aplysia californica* | 1632 | 9E-56 | 72 | 191 |  |
| Unigene0020434 | | CREB-binding protein | *Crassostrea gigas* | 4805 | 3E-120 | 46 | 426 |  |
| Unigene0015991 | | calcium/calmodulin-dependent protein kinase type II delta chain-like isoform X10 | *Crassostrea gigas* | 5757 | 0 | 88 | 883 |  |
| Unigene0038521 | | vascular endothelial growth factor receptor 1-like isoform X3 | *Crassostrea gigas* | 2432 | 2E-52 | 45 | 206 |  |
| Unigene0085442 | | PREDICTED: glucose transporter type 1-like | *Crassostrea gigas* | 1411 | 2E-154 | 61 | 458 |  |
| Unigene0037732 | | PREDICTED: cytochrome b-245 heavy chain-like | *Aplysia californica* | 1825 | 0 | 62 | 769 |  |
| Unigene0068109 | | aryl hydrocarbon receptor nuclear translocator | *Haliotis diversicolor* | 6528 | 0 | 98 | 1115 |  |
| Unigene0031035 | | PREDICTED: egl nine homolog 1-like isoform X2 | *Crassostrea gigas* | 6119 | 5E-114 | 46 | 378 |  |
|  | |  |  |  |  |  |  |  |

Note: Nr-Score, E value, ident were the Score, E-value and identity of Unigenes Blasts with Nr-database

Table S3

The 41 immune-related genes that were identified in transcriptome analysis results as DEGs and were confirmed by measuring their relative mRNA levels using qRT-PCR.

| **Unigene-Id** | **Gene name** | **Specific pathway** |
| --- | --- | --- |
| Unigene0027124 | PREDICTED: mitogen-activated protein kinase 14-like | MAPK signaling pathway |
| Unigene0004211 | PREDICTED: ribosomal protein S6 kinase alpha-5-like | MAPK signaling pathway |
| Unigene0032860 | Ras-related C3 botulinum toxin substrate 1, partial | MAPK signaling pathway |
| Unigene0038903 | PREDICTED: TGF-beta-activated kinase 1 and MAP3K7-binding protein 1-like | MAPK signaling pathway |
| Unigene0011922 | PREDICTED: mitogen-activated protein kinase kinase kinase 5-like | MAPK signaling pathway |
| Unigene0011448 | PREDICTED: MAP kinase-activated protein kinase 2-like | MAPK signaling pathway |
| Unigene0057926 | PREDICTED: TNF receptor-associated factor 2-like | MAPK signaling pathway |
| Unigene0058755 | PREDICTED: serine/threonine-protein phosphatase 5 isoform X2 | MAPK signaling pathway |
| Unigene0007411 | Protein max | MAPK signaling pathway |
| Unigene0066543 | PREDICTED: myocyte-specific enhancer factor 2A-like isoform X2 | MAPK signaling pathway |
| Unigene0019234 | MAP kinase kinase 3/6-like protein | MAPK signaling pathway |
| Unigene0035310 | Death domain-associated protein 6 | MAPK signaling pathway |
| Unigene0008684 | Serine/threonine protein kinase NLK | MAPK signaling pathway |
| Unigene0002072 | PREDICTED: MAP kinase-activated protein kinase 5-like | MAPK signaling pathway |
| Unigene0020490 | apoptosis regulator BAX-like | p53 signaling pathway |
| Unigene0023732 | Poly [ADP-ribose] polymerase 14 | p53 signaling pathway |
| Unigene0027926 | PREDICTED: caspase-7-like | p53 signaling pathway |
| Unigene0022793 | PREDICTED: caspase-2-like | p53 signaling pathway |
| Unigene0018770 | PREDICTED: E3 ubiquitin-protein ligase SIAH1-like | p53 signaling pathway |
| Unigene0000859 | cytochrome c | p53 signaling pathway |
| Unigene0017637 | PREDICTED: quinone oxidoreductase PIG3-like isoform X2 | p53 signaling pathway |
| Unigene0023472 | PREDICTED: caspase-3-like | p53 signaling pathway |
| Unigene0057698 | caspase 8 | p53 signaling pathway |
| Unigene0073521 | PREDICTED: caspase-6-like | p53 signaling pathway |
| Unigene0068266 | PREDICTED: apoptosis-stimulating of p53 protein 2-like | p53 signaling pathway |
| Unigene0031795 | myeloid differentiation factor 88 | p53 signaling pathway |
| **Unigene-Id** | **Gene name** | **Specific pathway** |
| Unigene0037728 | caspase 8 | p53 signaling pathway |
| Unigene0032372 | PREDICTED: phosphatidylinositol 4,5-bisphosphate 3-kinase catalytic subunit beta isoform-like | PI3K-AKT signaling pathway |
| Unigene0010626 | Eukaryotic translation initiation factor 4B | PI3K-AKT signaling pathway |
| Unigene0025341 | PREDICTED: eukaryotic translation initiation factor 4E type 2-like isoform X2 | PI3K-AKT signaling pathway |
| Unigene0024099 | PREDICTED: focal adhesion kinase 1-like isoform X3 | PI3K-AKT signaling pathway |
| Unigene0004729 | PREDICTED: hamartin-like isoform X2 | PI3K-AKT signaling pathway |
| Unigene0037961 | PREDICTED: tuberin isoform X1 | PI3K-AKT signaling pathway |
| Unigene0020620 | ikk-like protein | PI3K-AKT signaling pathway |
| Unigene0019283 | serine/threonine-protein kinase mTOR | PI3K-AKT signaling pathway |
| Unigene0021493 | PREDICTED: GTP-binding protein Rheb-like | PI3K-AKT signaling pathway |
| Unigene0095996 | PREDICTED: RAC-gamma serine/threonine-protein kinase-like | PI3K-AKT signaling pathway |
| Unigene0048947 | Fas ligand-like protein | PI3K-AKT signaling pathway |
| Unigene0021115 | PREDICTED: integrin-linked protein kinase-like isoform X2 | PI3K-AKT signaling pathway |
| Unigene0016439 | PREDICTED: toll-like receptor 4 | Toll-like receptor signaling pathway |
| Unigene0085234 | PREDICTED: nuclear factor NF-kappa-B p105 subunit-like isoform X2 | NF-kB signaling pathway |

Note: The false discovery rate (FDR) and absolute log2 Fold Change were used to screen the DEGs, and the screening criteria was set as FDR<0.05 and |log2FC|>1 by edgeR（An R software package for processing significant differences between paired samples）

Table S4**Primers used for RNAi in this study**

| **Primer name** | **Nucleotide sequence (5'→3')** | **Purpose** |
| --- | --- | --- |
| AKT-RNAi-F | TCTGCTCTTGGTTACCTGCA | RNAi |
| AKT-RNAi-R | ATACATGACCACTCCCGTCC | RNAi |
| GFP-RNAi-F | CGACGTAAACGGCCACAAGT | RNAi |
| GFP-RNAi-R | CTTCTACAGCTCGTCCATGC | RNAi |

Table S5 **Primers used for qRT-PCR in this study**

| **Primer name** | **Nucleotide sequence (5'→3')** |
| --- | --- |
| PI3K-RT-F | AGGCTGGGTAACGAATGTTG |
| PI3K-RT-R | GACTTCCGCTCTCGTTTGAC |
| AKT-RT-F | TCTGCTCTTGGTTACCTGCA |
| AKT-RT-R | ATACATGACCACTCCCGTCC |
| AKTIP-RT-F | GGCCGTTCTTCCAGGAGTAT |
| AKTIP-RT-R | CGGGGATGGTGAGTGTGAAC |
| TSC2-RT-F | CCGCCACCTGTAGATGAGAA |
| TSC2-RT-R | GCACTCCCGCTTGTTGTTAT |
| TSC1-RT-F | ACACACCCTCAGCATGATCA |
| TSC1-RT-R | TGATGCGCATGGACAATCTG |
| β−catenin-RT-F | GCAGCTAGCTCACAAAGCAC |
| β−catenin-RT-R | GCACTTCACATTCTTGCCCG |
| RHEB-RT-F | TTGTTCCCTTCATGTCCAGG |
| RHEB-RT-R | AGTTTGCCCTTCAGCTTGTG |
| mTOR-RT-F | ACAGGACGCCATTAAGACCA |
| mTOR-RT-R | TGTGGTGGGGATATGCTTGT |
| ILK-RT-F | AGGAGCTGGCTAGGCTACAA |
| ILK-RT-R | ATCTTGCCATCTACCACGCC |
| IKK-RT-F | TATTGTCCTGCAGCAAGTGG |
| IKK-RT-R | CTCTGGGATGCAAAAAGCTC |
| FASLG-RT-F | GTCGGCATATTTGCGACCAC |
| FASLG-RT-R | ACCGACGATCTTCAACACCC |
| FAK-RT-F | CTTAGTAAGACGGCGCCAAG |
| EIF4E-RT-F | CAACCATGTACGGAAGGCCA |
| FAK-RT-R | GGTGCTCTGGTACACGGAAT |
| EIF4E-RT-R | TGAGACTAATGAGCCAGCGG |
| EIF4B-RT-F | GGCGTCTACGAGGATTTGGT |
| EIF4B-RT-R | CTGTCCCGCCAAATCAACAC |
| β-actin-F | CCGTGACCTTACAGACTACCT |
| β-actin-R | TACCAGCGGATTCCATAC |
| P38-F | ACTGTCGAGGAAAGGGGAAT |
| P38-R | CGAATGTATTTCCGGGTTTG |
| **Primer name** | **Nucleotide sequence (5'→3')** |
| MSK1-F | CAGGCTCAGAGTCGAGACG |
| MSK1-R | GACATTGTCAACCGCTTCCT |
| RAC1-F | AACTTTGCGACAGCCATTTT |
| RAC1-R | CCAACAGCTCCGTCTCCTAC |
| TAB1-F | TGACCTGCCAGTGTGCTATC |
| TAB1-R | CGTCATCGGTTGTCTTGTTG |
| MAPKAPK2-F | TCCATCCAGACATCCTCCTC |
| MAPKAPK2-R | CCGAAATGGGCAGTATGAGT |
| TRAF2-F | GCACGGAGGAAGTTGCTAAG |
| TRAF2-R | AACTTCCCCTTCCATCTGCA |
| ASK1-F | CGCTTAGATGTGCCGAAGTC |
| ASK1-R | TGAGACGACCATTGCCTTCT |
| PPP5C-F | AGATGACTACACTGGCCCTG |
| PPP5C-R | TGTGATGTCAACTAAAGATGGCT |
| MAX-F | CTGCAGCTTTGTTCATGCCT |
| MAX-R | GCCCATATTTGAGTCAGGCC |
| MEF2C-F | GACCGTTTCATCAGCCTGTC |
| MEF2C-R | CACCGGTACCTCAGTCAAGT |
| MKK3-F | GCTCCAAACATCAGCTCGAG |
| MKK3-R | TGAAGCTAGGGGATTTCGGG |
| DAXX-F | GCATATCCGAGAACTTGCGG |
| DAXX-R | ACAACGACGACAGGATGACT |
| NLK-F | CTGTTCGCATGTCCTCAAGG |
| NLK-R | ACCGACCTCCAGAGATCCTA |
| PRAK-F | AAGCCACTGTGAAGCTCTCT |
| PRAK-R | AGTAGAAGGGAGGGTAGCCA |
| bax-F | CTCCCAAATGACGATCTGGT |
| bax-R | GTCCAGTCTTTGCCCACATT |
| Apaf-1-F | AGACGCTGAAGAAGGACCTG |
| Apaf-1-R | CGTACCCTTTATTCCCGGTAG |
| CASP3-F | CACACGGTTGATGACTTTGG |
| CASP3-R | CCCTGCTTCAAGGACTTCTG |
| CASP9-F | GACTTCAGCAGGGCAAGAAC |
| CASP9-R | GGGACAAGATGACACACACG |
| siah1-F | CAGTGGACTGGGTGATGATG |
| siah1-R | GCTCATGATTGCTGACTGGA |
| cyct1-F | ACGCTCCTGCTTTTTCTTGA |
| cyct1-R | GACGCAAGACTGGACAACAA |
| pigs-F | TATCGCCGATCTTCCATTTC |
| pigs-R | GGTGGTCCCGAGAATCTGTA |
| CASP7-F | GCTTGCAGGCAAACCTAAAC |
| CASP7-R | GCCTCTGTTGGGATCCTGTA |
| **Primer name** | **Nucleotide sequence (5'→3')** |
| CASP10-F | CGGTACCACTGGACGAGTTT |
| CASP10-R | TCCTTCAGCAGCAGTTCCTT |
| CASP6-F | GATGGTGGCTACGATGAGGT |
| CASP6-R | TTCCCTTGTTCCACGTTACC |
| P53-F | GCTTAGCTCATCCGAGTTGG |
| P53-R | CCAATGGTCAAGTTCCTCGT |
| MYD88-F | AGCATCCCTCAGCTTTTCCC |
| MYD88-R | CCTGTGCGACAATTCCTCCT |
| Toll4-F | TGCTGCAGAGTGCAAAGACT |
| Toll4-R | GCTGTTGAAGCTCCTTGACC |
| CASP8-F | GAGATGCAGGAAGGGGTACA |
| CASP8-R | ATGCTGAGCAGGTCCTCTGT |
| NFKB-F | GACCTGCTGGACGAAACGTA |
| NFKB-R | ACTCTCTTGTGTGGCAGCAA |
