# Supplementary material for: Immunity-related genes and signaling pathways under hypoxic stresses in Haliotis diversicolor: a transcriptome analysis
Source: Sci Rep. 2019 Dec 24;9:19741. doi: 10.1038/s41598-019-56150-2 (PMC6930256; doi:10.1038/s41598-019-56150-2)
Supplement: Supplementary file 2 — Supplementary information 2 [file 41598_2019_56150_MOESM2_ESM.docx]

**Additional file 2:**

**Immunity genes and signaling pathways under hypoxic stresses in *Haliotis diversicolor*: a transcriptome analysis**

Yulong Sun^1,3^, Xin Zhang^1,3^, Yilei Wang^2^*, Robert Day^4^, Huiping Yang^5^, and Ziping Zhang^1,2^*

1 College of Animal Science, Fujian Agriculture and Forestry University, Fuzhou 350002, PR China

2 Fisheries College, Jimei University, Xiamen, 361021, China

3 Key Laboratory of Marine Biotechnology of Fujian Province, Institute of Oceanology, Fujian Agriculture and Forestry University, Fuzhou 350002, PR China

4 School of Biosciences, University of Melbourne, Parkville, Victoria, Australia 3010

5 School of Forest Resources and Conservation, IFAS, University of Florida, 7922 NW 71st Street, Gainesville, FL 32615


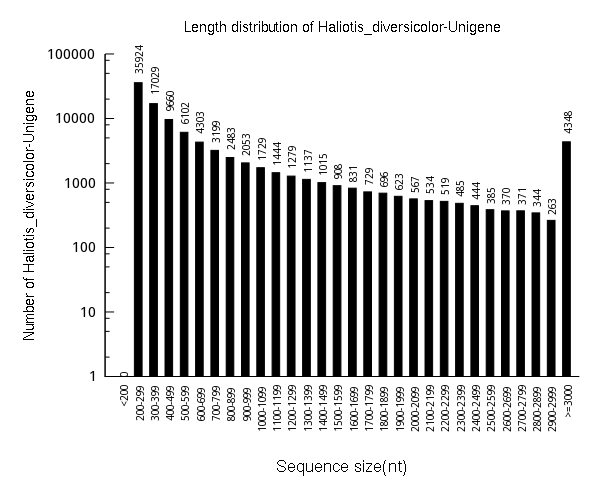


Fig. S1

The sequence size distribution of all unigenes in the transcriptomic profiles of *H. diversicolor.* The average length of a transcript was 768.27 bp and the N50 length was 1414 bp. The overall size range is from 200 to >3000 bp.


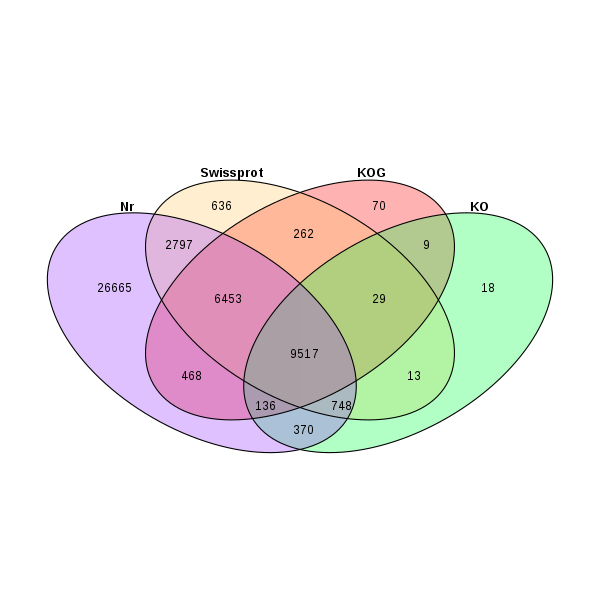


Fig. S2 Venn diagram of all the unigenes annotated against the Nr, SwissProt, COG and KEGG databases. The number in each color block indicates the number of unigenes that is annotated by single or multiple databases. A total of 48,191 unigenes were identified from 51,583 consensus sequences of *H. diversicolor* and 47,154 were identified in Nr and 20,455 in Swissprot. In addition, 16,944 and 10,840 unigenes could be classified by the KOG and KEGG databases, respectively.

Fig. S3 GO function classification of *H. diversicolor* unigenes. The 99,774 unigenes were classified in terms of GO functions by the Blast2GO software: 22,572 unigenes belong to the biological process category, 12772 to the cellular component and 11322 to the molecular function categories.

Fig. S4-A

Fig. S4-B

Fig. S4-C

Fig. S4-D

Fig. S4-E

Fig. S4-A-E GO functional classification of DEGs at all sampling times (4h, 12h, 24h and 48h).

The differences in expression between the treatment groups, the blank group (BC), normal condition (NC) and hypoxia stress (HS) were compared with each other. The ‘biological process’ classification was analysed further, and the detailed functional classifications in this category are shown.

A: the comparison of NC and BC at 4h, 12h (NC-4h/BC-4h; NC-12h/BC-12h).

B: the comparison of NC and BC at 24h, 48h (NC-24h/BC-24h; NC-48h/BC-48h).

C: the comparison of HS and NC at 0h, 4h (HS-0h/NC-0h; HS-4h/NC-4h).

D: the comparison of HS and NC at 12h, 24h (HS-12h/NC-12h; HS-24h/NC-24h).

E: the comparison of HS and NC at 48h (HS-48h/NC-48h).

Fig. S5 Heat map representation of the mRNA expression level of 225 immune-related genes in haemocytes at 4 h, 12 h, 24 h and 48 h post-injection, when *H. diversicolor* had been exposed to bacterial challenge (NC). The color scale at the far right of the heat map represents the RPKM relative expression value (log2 NC/BC), where red, green and black colors indicate up-regulation, down-regulation and unaltered expression, respectively, relative to the BC Control group.

Fig. S6 Heat map representation of the mRNA expression level of 225 immune-related genes in haemocytes at 4 h, 12 h, 24 h and 48 h post-injection, when *H. diversicolor* had been exposed to hypoxia stress and bacterial challenge (HS). The color scale at the far right of the heat map represents the RPKM relative expression value (log2 HS/NC), where red, green and black colors indicate up-regulation, down-regulation and unaltered expression, respectively, relative to the NC Control group.

Fig. S7 Trend analyses of changes between sample times in each treatment.

A：trend analysis I（BC-0h、BC-4h、BC-12h、BC-24h、BC-48h）B：trend analysis II（BC-0h、NC-4h、NC-12h、NC-24h、NC-48h）C：Trend analysis III（HS-0h、HS-4h、HS-12h、HS-24h、HS-48h. A total of 11813 unigenes were down-regulated, accounting for 48% of the total DEGs (trend analysis III).

BC: Blank control group, normal conditions; NC: Control group, bacterial challenge under normal conditions; HS: Experimental group, hypoxia stress and bacterial challenge. The colors represent different types of trends.

Fig. S8

The heat map of the RPKM relative expression of 41 immune-related genes under bacterial infection (Normal control group, NC) and hypoxia stress and bacterial challenge (Experimental group, HS).

A: The color scale at the far right of the heat map represents the RPKM relative expression value (log2 NC/BC), where red, green and black colors indicate up-regulation, down-regulation and unaltered expression, respectively, relative to the BC blank control group.

B: The color scale at the far right of the heat map represents the RPKM relative expression value (log2 HS/NC), where red, green and black colors indicate up-regulation, down-regulation and unaltered expression, respectively, relative to the NC normal control group.

Fig. S9-A

Fig. S9-B

Fig. S9-A & B: The expression fold change under hypoxia over time of 41 DEGs identified in transcriptome analysis using RPKM values and qRT-PCR. X axis: hours post bacterial injection (0 h, 4h, 24h and 48h). Y axis: fold change of expression in the experimental group (HS) as compared with the control group (NC) (HS/NC). Vertical bars show the fold change based on Real-time quantitative PCR results （HS/NC）. The significant difference between the experimental group (HS) and the control group (NC) is indicated by a (*) at *p* < 0.05. Lines show the fold change using the RPKM results (HS/NC). β-actin served as the reference gene for Real-time quantitative PCR. The patterns of expression for 27 unigenes were consistent between the two methods.

Fig. S10 The heat map of the RPKM values and qRT-PCR relative expression for 41 immune-related genes under hypoxia stress and bacterial challenge (Experimental group, HS).

A: The color scale at the far right of the heat map represents the RPKM relative expression value (log2 HS/NC), where red, green and black colors indicate up-regulation, down-regulation and unaltered expression, respectively, relative to the NC Control group.

B: The color scale at the far right of the heat map represents the relative mRNA expression level (log2 HS/NC), where red, green and black colors indicate up-regulation, down-regulation and unaltered expression, respectively, relative to the NC Control group.
